# Genomic signals of local adaptation across climatically heterogenous habitats in an invasive tropical fruit fly (*Bactrocera tryoni*)

**DOI:** 10.1038/s41437-023-00657-y

**Published:** 2023-10-30

**Authors:** Elahe Parvizi, Amy L. Vaughan, Manpreet K. Dhami, Angela McGaughran

**Affiliations:** 1 Te Aka Mātuatua/School of Science, University of Waikato, Hamilton, New Zealand; 2 Biocontrol and Molecular Ecology, Manaaki Whenua Landcare Research, Lincoln, New Zealand

**Keywords:** Genetic variation, Population genetics, Invasive species

## Abstract

Local adaptation plays a key role in the successful establishment of pest populations in new environments by enabling them to tolerate novel biotic and abiotic conditions experienced outside their native range. However, the genomic underpinnings of such adaptive responses remain unclear, especially for agriculturally important pests. We investigated population genomic signatures in the tropical/subtropical Queensland fruit fly, *Bactrocera tryoni*, which has an expanded range encompassing temperate and arid zones in Australia, and tropical zones in the Pacific Islands. Using reduced representation sequencing data from 28 populations, we detected allele frequency shifts associated with the native/invasive status of populations and identified environmental factors that have likely driven population differentiation. We also determined that precipitation, temperature, and geographic variables explain allelic shifts across the distribution range of *B. tryoni*. We found spatial heterogeneity in signatures of local adaptation across various climatic conditions in invaded areas. Specifically, disjunct invasive populations in the tropical Pacific Islands and arid zones of Australia were characterised by multiple significantly differentiated single nucleotide polymorphisms (SNPs), some of which were associated with genes with well-understood function in environmental stress (e.g., heat and desiccation) response. However, invasive populations in southeast Australian temperate zones showed higher gene flow with the native range and lacked a strong local adaptive signal. These results suggest that population connectivity with the native range has differentially affected local adaptive patterns in different invasive populations. Overall, our findings provide insights into the evolutionary underpinnings of invasion success of an important horticultural pest in climatically distinct environments.

## Introduction

Spatial variation in environmental factors between native and introduced species’ ranges can often drive local adaptation in non-native populations (Sotka et al. 2018; van Boheemen et al. 2019) which facilitates rapid range expansion across environmentally different habitats (Colautti and Barrett 2013). For insects in particular, variations in behavioural (e.g. migratory traits, Jones et al. 2015), phenotypic (e.g. morphotypes, Yadav et al. 2018), life history (e.g. reproductive modes, Sandrock et al. 2011) and physiological traits (e.g. temperature tolerance, Fält-Nardmann et al. 2016) have emerged in response to varying environmental conditions. Identifying the molecular signatures underlying such local adaptive changes remains a challenge, particularly for species with limited genomic resources, as this requires high-density polymorphism data and functionally annotated genomes – which are often lacking (Matheson and McGaughran 2022). However, availability of insect pest genomes is increasing with the affordability of high-throughput sequencing, leading to new insights on the genomic characteristics that contribute to the adaptive potential of invaders (Pélissié et al. 2018). Climate warming is compounding the severity of the threats imposed by insect pests on native and productive systems, e.g., by increasing geographic expansion rates and survival during overwintering, and/or altering interspecific interactions (Barbet-Massin et al. 2013; Hulme 2017; Skendžić et al. 2021). Thus, genomic insights are crucial for determining invasion success more broadly in novel environments, as well as for providing new knowledge about long-term effects and sustainability of different pest control strategies (Sethuraman et al. 2020).

Identifying evidence of natural selection acting on genomic diversity is a key step in understanding local adaptive processes that underpin invasion success. For local adaptation to occur, natural selection must be stronger than the homogenising effect of inter-population gene flow and random genetic drift (Kawecki and Ebert 2004). Specifically, gene flow is an important evolutionary force that can interfere with local adaptation through the exchange of alleles among populations from divergent environments (Sexton et al. 2014), and the homogenisation of locally adapted alleles under weak selection (Lenormand 2002; Akerman and Bürger 2014). Alternatively, gene flow can promote local adaptation by introducing novel genetic variation or pre-adapted alleles into environmentally variable populations (Tigano and Friesen 2016; Pfeifer et al. 2018; Zhang et al. 2021; Montejo-Kovacevich et al. 2022). This contrasting role of gene flow in constraining or promoting local adaptation is particularly important for invasive species that occupy environmentally different habitats and are often subject to repeated human-mediated introductions (Smith et al. 2020; Yadav et al. 2019). For instance, the bridgehead effect – repeated gene flow from source to expanded ranges or among expanded ranges – is a key driver of successful insect invasions (e.g. Eyer et al. 2021; Javal et al. 2019; Parvizi et al. 2022). However, the extent to which gene flow between native and expanded ranges could affect the genomic signatures of local adaptation in invasive pest populations remains understudied.

**Fig. 1:**
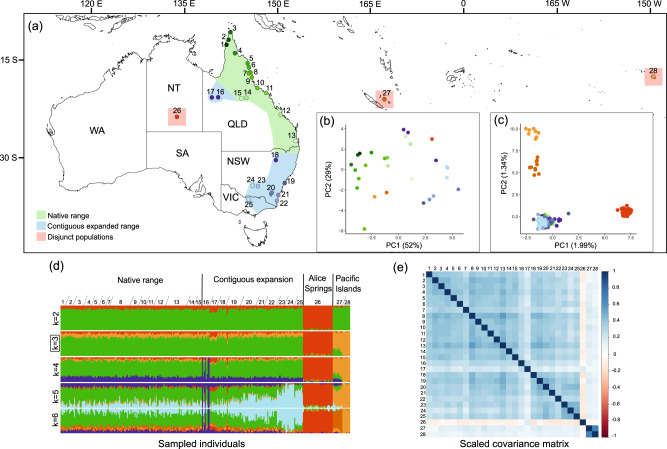
Sampling sites and genetic structure of *Bactrocera tryoni*. **a** Geographic location of native and invasive populations. The expansion of *B. tryoni* in southern regions started ~150 years ago, while incursions further inland in Queensland (sites 16 and 17) and southeastern Australia happened later (around 1994, Osborne et al. 1997; Popa-Báez, Catullo et al. 2020). The first record of *B. tryoni* in central Northern Territory (site 26) was in 1987 (Cameron 2006), in the Loyalty Islands (site 27) was around 1969, and in French Polynesia (including Tahiti Island; site 28) was a year later (1970) - a pattern consistent with a succession of ‘island hopping’ invasions (Popa-Báez, Catullo et al. 2020). The geographic distribution of native and different invasive ranges follows the suggested ranges proposed by Popa-Báez, Catullo et al. (2020). NT: Northern Territory, QLD: Queensland, NSW: New South Wales, VIC: Victoria, SA: Southern Australia, WA: Western Australia; **b** Principal components analysis (PCA) of 19 bioclimatic variables showing the extent of environmental differentiation among different populations; **c** PCA showing SNP variation among individuals across native and different invasive populations; **d** Admixture plots of assignment probabilities for k = 2–6 clusters for each individual inferred from sparse non-negative matrix factorisation (sNMF) analysis. Each vertical bar represents an individual and colours represent admixture proportions. K = 3 was the most optimal clustering pattern based on a cross-entropy criterion (see Fig. S2); **e** Correlation plot showing population scaled covariance matrix (Ω) obtained with the BayPass core model indicating genetic structuring in invasive populations. Note that the PCA, sNMF and Ω matrix plots are based on analysis of 6707 SNPs in 301 individuals.

The Queensland fruit fly, *Bactrocera tryoni* (Diptera: Tephritidae) is a major pest native to wet subtropical/tropical coastlines of eastern Australia including Queensland and northern New South Wales (Fig. 1a, b; May 1963; Popa-Báez, Catullo et al. 2020). With the development of horticultural industries over the past ~150 years, *B*. *tryoni* moved from native host plants to cultivated fruit, contiguously expanding its range southwards into temperate areas of New South Wales and northern Victoria (O’loughlin et al. 1984; Dominiak and Daniels 2012; Popa-Báez, Catullo et al. 2020). Genome-wide SNP data has revealed a high degree of homogeneity between *B*. *tryoni* in the native and contiguous expansion populations (Popa-Báez et al. 2020). Additionally, some disjunct and genetically isolated populations have been identified in arid parts of the central Northern Territory as well as from subtropical/tropical South Pacific Islands (Clarke et al. 2011; Popa-Báez, Catullo et al. 2020). This successful spatial expansion of *B*. *tryoni* into climatically diverse regions suggests that the species has a high capacity for local adaptation. Additionally, varying levels of genetic connectivity between different invaded areas and the native range (Popa-Báez, Catullo et al. 2020) make *B. tryoni* an ideal species for understanding the effects of native-introduced range connectivity in shaping local adaptation patterns.

Throughout the expanded range of *B*. *tryoni*, survival and reproduction are largely influenced by temperature, moisture, and the presence of suitable host fruits (Meats 1984; Sutherst and Yonow 1998), with lower temperature and desiccation stress restricting further range expansion in the south (Sutherst and Yonow 1998; Sultana et al. 2017). Common garden experiments have identified significant differences in heat and desiccation resistance between different *B*. *tryoni* populations, with invasive populations in arid and temperate regions exhibiting higher heat and desiccation resistance, respectively (Popa-Báez, Lee et al. 2020). Nevertheless, as these two traits did not show correlation or clinal variation (Popa-Báez, Lee et al. 2020), complex patterns of local adaptation across native and expanded ranges are expected.

Loci underpinning local adaptation can be identified by applying genome scan methods that detect shifts in allele frequency or genomic variants with higher than expected divergence among populations, while accounting for neutral demographic history (Whitlock and Lotterhos 2015; Olazcuaga et al. 2020). Specifically, invasion-associated adaptive genomic variants can be identified by contrasting allele frequencies between native and invasive populations (e.g. C2 statistic; Olazcuaga et al. 2020). Further, the non-linear association of adaptive loci to spatial and environmental variables, and therefore patterns of allele frequency shifts among geographic locations and across environmental gradients (i.e. allelic turnover), can be examined using turnover functions, such as the gradient forest approach (Fitzpatrick and Keller 2015). Here, we combine genome-wide scanning with spatial population structure modelling and gradient forest analysis across native and invasive ranges of *B*. *tryoni* using the previously published population genomic dataset of Popa-Báez, Catullo et al. (2020) to investigate: (i) genomic signatures of local adaptation associated with invasive status of populations; (ii) the influence of connectivity between the native range and the invasive range on observed genomic patterns of local adaptation; and (iii) the relative importance of environmental variables to allele frequency changes at neutral and adaptive loci. Our results reveal complex patterns of local adaptation in contiguous versus disjunct invasive populations, with spatially explicit adaptive genetic divergence in isolated populations. These insights have implications for understanding future adaptive responses of this and other insect pests during the invasion of heterogenous environments.

## Materials and methods

### Population sampling and variant calling

We analysed Diversity Arrays Technology (DArT) sequencing data generated by a previous population genomic study on *B. tryoni* by Popa-Báez, Catullo et al. (2020). While Popa-Báez, Catullo et al. (2020) identified spatial and temporal patterns of genome-wide diversity and differentiation in *B. tryoni*, we here use the same dataset to further explore local adaptation, gene flow and the geographic distribution of genomic variation along environmental gradients. Briefly, the DArT sequencing approach included the digestion of DNA using *PstI* and *SphI* restriction enzymes, ligation of <200 bp barcode adapters to the two restriction enzyme overhangs, amplification of fragments using PCR, and sequencing on the Illumina Hiseq 2500 (Georges et al. 2018; Popa-Báez, Catullo et al. 2020). Our dataset included 301 *B*. *tryoni* individuals sampled from 15 native populations across the east coast of Queensland and 13 introduced populations from eastern New South Wales, eastern Victoria, north-western Queensland, central Northern Territory and two Pacific Islands (Fig. 1a, Table S1).

We used the BWA MEM v. 0.7.17 algorithm (Li and Durbin 2009) to map sequence reads from each individual to the *B*. *tryoni* chromosome-level reference genome, CSIRO_BtryS06_freeze2 (available at NCBI under BioProject number PRJNA560467), using default parameters and the *M* and *R* flags to mark secondary reads and add read groups, respectively. We converted the resulting sequence alignment map (SAM) files to sorted binary alignment map (BAM) files and marked duplicate reads using SAMtools v. 1.15.1 (Li et al. 2009). To call variants, we used *mpileup* and *call* modules of BCFtools v. 1.13 (Li 2011), using the default calling method, outputting only variant positions, and including variants with minimum mapping quality and minimum base quality scores of 20, and excluding those with read depth <10X or >250X. Using BCFtools, we also removed all indels, non-biallelic SNPs, and SNPs with a minimum allele frequency (MAF) < 0.05. We used PLINK v. 2 (Chang et al. 2015) to hard-filter genotypes, removing SNPs with high missing rates (> 10%) and high correlation with other SNPs within 50 kb windows (r^2^ > 0.2).

### Spatial genomic diversity and structure

We used the *populations* module of Stacks v. 2.58 (Rochette et al. 2019) to calculate overall nucleotide diversity (π), average observed and expected heterozygosity, and inbreeding coefficient (F_is_) for each local population. We tested for significant differences in these population genomic indices between native and invasive ranges using t-tests or the non-parametric Wilcoxon rank-sum test. We investigated population structure across native and expanded ranges by performing a principal component analysis (PCA) using the R package *adegenet* v. 2.1.1 (Jombart 2008) and calculating pairwise F_st_ between each local population with 100 bootstraps in the R v. 4.1.1 (R Core Team 2021) package *StAMPP* v. 1.5.1 (Pembleton et al. 2013). We investigated patterns of linkage disequilibrium (LD) decay across SNPs in populations with *n* > 5 with PopLDdecay v. 3.42 (Zhang et al. 2019), using default parameters except changing the maximum distance between two SNPs to 1 kb to account for the lack of dense SNPs obtained from DArT sequencing. PopLDdecay was run on the dataset that was not pruned for highly correlated SNPs.

We performed a non-negative matrix factorisation (sNMF) analysis using the R package *LEA* v. 2.8.0 (Frichot et al. 2014; Frichot and François 2015) to explore spatial structure and estimate individual ancestry coefficients for values of k ranging from 1–10. We ran the sNMF analysis with 50 iterations of the algorithm per k-value and plotted the cross-entropy results for each k-value to identify the optimal clustering level. Using the R package *pophelper* v. 2.3.1 (Francis 2017), we plotted admixture bar plots for k = 2–10 to visually explore hierarchical population structure across the studied area. Additionally, to investigate spatial genomic structure within the introduced ranges of *B*. *tryoni*, we repeated the sNMF analysis on a dataset excluding samples from the native range. We also explored covariance structure among population allele frequencies resulting from their shared demographic histories (Olazcuaga et al. 2020) by estimating the scaled covariance matrix of the population allele frequencies (Ω) using the BayPass v. 2.3 core model (Gautier 2015).

### Patterns of gene flow, isolation and discrete population structure

We estimated effective migration surfaces across the studied area to highlight regions with higher-than-average and lower-than-average historical gene flow using the programme EEMS (Petkova et al. 2016). EEMS implements the concept of isolation by resistance (McRae 2006), which characterises variation in migration rates between adjacent locations in a stepping-stone model, to identify barriers to gene flow across the landscape (Petkova et al. 2016). By using geographically indexed genetic data, EEMS evaluates how genetic similarity decays with geographic distance and highlights areas that deviate from exact isolation by distance (Petkova et al. 2016). We generated a full-rank genetic distance matrix between each individual by imputing missing genotypes with observed mean genotypes at each SNP using the bed2diffs_v1 R function (distributed with the EEMS software). We produced a habitat polygon based on the geographic distribution of our sampling sites, excluding the Tahiti Island population to obtain an enclosed polygon around Australia. The habitat polygon was created using the Google Maps API v3 tool (http://www.birdtheme.org/useful/v3tool.html). We distributed 200 demes over the habitat polygon and performed two independent Markov chain Monte Carlo runs for 5 million generations with the first 2 million generations excluded as burn-in. We visually assessed the convergence of the chains and combined the runs to generate maps of effective migration surfaces and effective genetic diversity using the R package reemsplot (Petkova et al. 2016).

To identify whether genetic divergence is represented as discrete clusters or continuous clines, we used the R package conStruct v. 1.0.5 (Bradburd et al. 2018). ConStruct simultaneously tests for continuous and discrete population structure by estimating ancestry proportions for each sampled individual from two-dimensional population layers, where a rate at which relatedness decays with distance is inferred within each layer (Bradburd et al. 2018). We created a matrix of allele frequency data for all our sampled individuals using the *structure2conStruct* function of the R package conStruct. A matrix of pairwise geographic distances between all samples was created by calculating pairwise great-circle distance between sampling coordinates using the *rdist.earth* function of the R package fields v. 14.1 (Nychka et al. 2021). We tested for the presence of k = 1–6 clusters by comparing the spatial (which accounts for geographic distance between samples) and non-spatial models. For each k value, we used 90% of the data to train both models and performed three replicates with 5,000 iterations per replicate. We compared the spatial and non-spatial models using cross-validation and chose the models with the highest value of predictive accuracy (i.e., close to zero) as the best model (Bradburd 2023). To identify the optimal k value associated with the best model, we calculated the total covariance contribution for each k within the same model. We set an arbitrary threshold criterion of 0.02 for the covariate contribution, selecting the k value that fell above this cutoff as the most suitable (Bradburd 2023).

### Local and shared adaptive loci

Spatial genomic analysis showed the presence of three clusters within the introduced ranges of *B*. *tryoni*: an Alice Spring cluster, a Pacific Islands cluster, and a contiguous expanded cluster (see Results). To identify outlier SNPs in each of these clusters, and test for shared adaptive processes that may be acting across the introduced ranges of *B*. *tryoni*, we used BayPass v. 2.3 (Gautier 2015; Olazcuaga et al. 2020). BayPass accounts for confounding effects of shared demographic history and population structure when identifying variants whose allele frequencies deviate significantly from the multivariate normal distribution (Gautier 2015). BayPass also allows users to specify binary covariates to test for association with genomic variants (Olazcuaga et al. 2020). Here, we used the invasion status of populations as a covariate to identify SNPs that showed significant allele frequency differences between native and introduced populations by estimating the contrast statistic, C_2_ (Olazcuaga et al. 2020).

We ran the BayPass core model with default parameters to contrast 15 Queensland native populations to all 13 invasive populations. Additionally, we identified signals common or specific to each invasion cluster (Olazcuaga et al. 2020) by separately contrasting native populations to the Alice Springs, Pacific Islands, and remaining contiguous expanded populations. The BayPass C2 statistic approach has been suggested to be robust when dealing with datasets that include a small number of populations (e.g., <8 populations) as the C2 estimation does not require the inclusion of any model parameters (Olazcuaga et al. 2020). We applied a false discovery rate of 5% using the R package qvalue v. 2.26.0 (Storey and Tibshirani 2003) and chose SNPs with q-value < 0.05 as the best adaptive candidates in different introduced ranges of *B*. *tryoni*. We also assessed the convergence and reproducibility of MCMC estimates by repeating each BayPass run three times using different seeds. Using the R package ggvenn v. 0.1.9 (Yan 2021), we created a Venn diagram to explore shared candidate SNPs among different introduced clusters.

### Functional annotation of candidate genes

To investigate genes associated with putatively adaptive loci identified with BayPass, we used BEDTools v. 2.29.2 to extract flanking sequence position information at 10 kb distance from outlier SNPs. We then used the UCSC Table Browser (https://genome.ucsc.edu/; last accessed November 2022) to retrieve the transcript IDs of the exons and coding DNA sequences located at these flanking regions. To identify gene IDs and predicted proteins in *B*. *tryoni*, we checked each transcript ID against the NCBI nucleotide database. We also searched for the putative function of the candidate genes by comparing them with homologous genes in the *Drosophila melanogaster* database (https://flybase.org/, last accessed November 2022) or other species.

To formally assess the gene functions related to putatively adaptive loci, we performed a Gene Ontology (GO) enrichment test using the R package topGO v. 2.4 (Alexa and Rahnenfuhrer 2022). We used InterProScan v. 5.51 (Jones et al. 2014) to initially get the genomic GO annotation for all loci (candidate and non-candidate) and create a complete set of GO terms as a reference for the enrichment analysis. Then, using the weight01 algorithm and Fisher’s exact test of topGO, we identified GO terms that were significantly over-represented in our candidate adaptive gene set (*p* value < 0.05) for molecular functions and cellular components.

### Environmental correlates of genomic variation

To investigate the relative importance of predictor variables (including climatic and spatial variables) in explaining allele frequency changes across the native and introduced ranges of *B*. *tryoni*, we performed gradient forest (GF) analysis (Ellis et al. 2012; Fitzpatrick and Keller 2015). Gradient forest is a non-parametric, machine learning regression tree approach initially developed to study ecological community turnover along environmental gradients by creating individual models for each species and identifying community turnover via aggregating (averaging) environmental predictor importance for each species (Ellis et al. 2012). Fitzpatrick and Keller (2015) extended this concept to landscape genomics, where allele frequency at genetic loci replace species, and the focus is on determining how well the environmental factors explain the variance in allele frequencies. This approach allows for the identification of the environmental variables that drive observed changes in allele frequency and the structuring of genetic variation (Fitzpatrick and Keller 2015). GF analysis can be applied to large genomic datasets, either on their own or in conjunction with first conducting scans for local adaptation using outlier detection or gene-environment association approaches (Fitzpatrick and Keller 2015). Furthermore, it can be used to model the functional turnover of neutral and adaptive diversity along environmental gradients by incorporating neutral and adaptive SNPs, respectively (e.g., Cao et al. 2021).

We obtained 19 bioclimate variables in raster format at a spatial resolution of 30 seconds, available from the WorldClim2 database (Fick and Hijmans 2017). We extracted climate data for each sampling site of *B*. *tryoni* from these raster files using the R package raster v. 3.5–29 (Hijmans et al. 2022). We excluded climate variables with Pearson’s correlation coefficient >0.6 to other variables, eventually retaining six variables including bio_3 (isothermality, calculated as the ratio of the mean diurnal temperature range to the annual temperature range, multiplied by 100), bio_5 (maximum temperature of warmest month), bio_8 (mean temperature of wettest quarter), bio_9 (mean temperature of driest quarter), bio_12 (annual precipitation), and bio_19 (precipitation of coldest quarter). To define spatial variables, we used principal coordinates of neighbourhood matrices (PCNMs) based on geographic coordinates using the *pcnm* function of the R package vegan v. 2.6–2 (Oksanen et al. 2022).

We conducted the GF analysis using population-based allele frequencies because several individuals were genotyped at each locality and thus experienced the same environmental conditions (Capblancq and Forester 2021). To ensure small sample size (*n* < 5) did not affect population allele frequency estimates, we removed Weipa (*n* = 5) and Mareeba (*n* = 4) from the GF analysis. To calculate population allele frequencies for each sampling site and impute missing genotypes, we followed the procedure recommended in Capblancq and Forester (2021). Briefly, we first created a genotype matrix with individuals in rows and SNPs in columns using the *extract.gt* function of the R package vcfR v. 1.12.0 (Knaus and Grünwald 2017). We then applied the *aggregate* function of R to the genotype matrix to calculate mean allele frequency per population, replacing missing genotypes with the median locus allele frequencies across each population.

We used the R package gradientForest v. 0.1–32 (Smith and Ellis 2021) to fit GF models, using environmental and spatial data as predictors. We modelled the turnover of both neutral and adaptive genetic variation across the landscape. For the neutral variation, we used all SNPs except for the BayPass outliers. For the adaptive variation, we specifically included the BayPass outliers, including those detected from comparing the native range versus each invasive lineage as well as the native range versus pooled invasive dataset. GF analysis was performed using 1,000 regression trees per SNP. We set the variable correlation threshold at 0.5 and used the default values for the proportion of samples used for training (0.632) and testing (0.368) for each tree. We visually represented the changes in allele frequencies along spatial and environmental gradients by plotting the aggregate turnover functions obtained from the GF analysis.

## Results

### Population genomic diversity, structure, and gene flow

Our final hard-filtered dataset included 6,707 SNPs from 301 *B*. *tryoni* samples across 15 native and 13 introduced populations. We found significant differences in nucleotide diversity (*t* = 4.336, *p* value = 0.0003) as well as expected heterozygosity (W = 148, *p*-value = 0.019) between the native and introduced range of *B*. *tryoni*. However, observed heterozygosity showed no significant difference between the two ranges (*t* = 0.057389, *p*-value = 0.95). Inbreeding coefficients were slightly higher in native range (mean F_is_ = −0.09) compared to the introduced range (mean F_is_ = −0.11), and these differences were statistically significant (W = 162, *p*-value = 0.002). Table S2 summarises the details of each population genomic index. We found broadly consistent patterns of LD decay across native and invasive populations (Fig. S1).

In the PCA, all native populations were clustered with the contiguous invasive populations in western Queensland, eastern New South Wales, and Victoria, while the disjunct invasive populations of Alice Springs and Pacific Islands formed highly distinct clusters (Fig. 1c). Pairwise F_st_ was comparatively lower in the native range (average 0.016), and Alice Springs and the two Pacific Islands showed the highest differentiation (average 0.04 and 0.05, respectively; Table S3). Consistent with this, we found high admixture between native and all expanded populations except Alice Springs and the Pacific Islands (Fig. 1d). The cross-entropy plot confirmed an optimal clustering of individuals into three groups (i.e., k = 3; Fig. S2). At higher k values, some populations from within contiguous expanded range, including the southernmost introduced populations and the inland (non-coastal) Queensland population in Cloncurry, showed more genomic differentiation (Figs. 1d; S2). When excluding the native population from the sNMF analysis, the introduced populations were optimally divided into three distinct clusters: the Alice Spring cluster, the Pacific Islands cluster, and the contiguous expanded populations in western Queensland, eastern New South Wales, and Victoria (hereafter regarded as the ‘contiguous expansion cluster’). The population scaled covariance matrix (Ω) identified structuring of genetic diversity across the populations, with strong differentiation of Alice Springs, the Pacific islands, Cloncurry, and Mount Isa relative to other native and introduced populations (Fig. 1e).

**Fig. 2:**
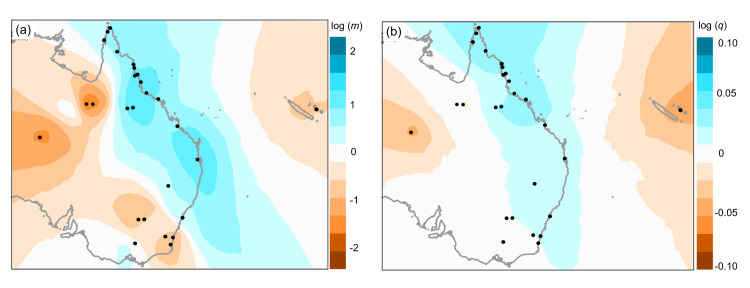
Spatial patterns of genetic connectivity and variability in *Bactrocera tryoni* produced by EEMS analysis. **a** Estimated migration patterns as posterior migration rates, log (*m*). Darker brown colours indicate reduced migration across the region, and darker blue colours indicate higher migration rate than expected; **b** Estimated diversity patterns as posterior diversity rates, log (*q*). Darker brown colours indicate lower diversity (genetic dissimilarity), and darker blue colours indicate higher diversity rates.

The migration surface estimated by EEMS indicated the highest genetic connectivity within the native range (Fig. 2a). Comparing gene flow between the native range and different invasive ranges, EEMS identified higher gene flow with the contiguous expanded range compared to the disjunct invasive ranges. However, gene flow decreased towards the edges of the contiguous expanded range (both towards inland Queensland as well as southeastern Australia). The Alice Springs and Loyalty Islands populations were estimated to be highly isolated. EEMS also estimated that genetic diversity rates were highest closer to the native range and decreased towards the edges of the contiguous expanded ranges as well as in the disjunct Alice Springs and Loyalty Islands populations (Fig. 2b).

ConStruct’s cross-validation approach for model comparison found higher predictive accuracy for the spatial model over the non-spatial model over all tested values of k (Fig. S3a), implying that isolation by distance is probably a feature of the data (Bradburd 2023). The spatial cross-validation results showed almost equal support for the conclusion that k = 3, 5, and 6 are sufficient to describe the variation in the data. However, as these results can be affected by spurious statistical support for layers that contribute little to overall patterns of covariance (Bradburd 2023), we calculated layer contributions for the spatial model and chose the best k value considering layer contribution of > 0.02. Accordingly, we concluded that the best value of k for describing our data is no greater than three (Fig. S3b). At k = 3 for the spatial model, Tahiti Island appears as a discrete cluster, Alice Springs, Loyalty Islands, and the edge populations of the contiguous expanded range (both towards inland Queensland as well as southeastern Australia) form the second discrete cluster, and the rest of the contiguous expanded range along with the native range represented the third cluster. ConStruct indicated varying levels of admixture between all the three spatial clusters (Fig. S3c).

### Loci associated with population invasive status

**Fig. 3:**
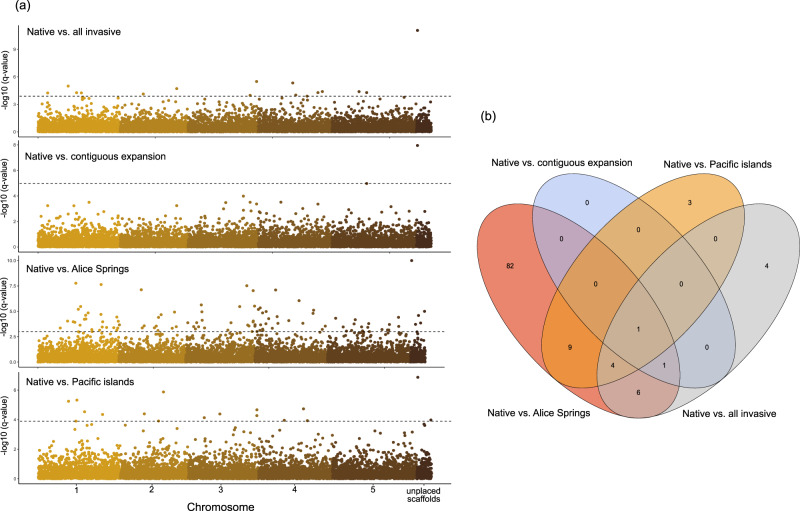
Genome-wide scan for association of SNPs with invasion status in *Bactrocera tryoni*. **a** Manhattan plots showing the distribution of SNPs (q values on a – log_10_ scale) associated with the invasive status of populations. These SNPs are derived from the C_2_ statistic, obtained by contrasting native versus different invasive clusters using BayPass. The horizontal dashed line indicates the 5% *q*-value threshold. **b** Venn diagram showing the number of shared and unique invasive-associated outlier SNPs in different invasive clusters.

Genome-wide scans using BayPass identified SNPs associated with the invasive status of populations within all introduced ranges of *B. tryoni* (Fig. 3a). Contrasting allele frequency changes between all native and invasive populations identified 16 outliers (Fig. 3b). Contrasting allele frequency changes between the native range and the contiguous expansion cluster identified two outliers, while contrasting Alice Springs and the Pacific Islands with the native range identified 103 and 17 outliers, respectively (Fig. 3b).

Using the *B. tryoni* genome assembly available at the UCSC Table Browser tool, we identified exons within 10 kb (up- or down-stream) of only 73 outliers (out of the total 110 outliers obtained from all the BayPass contrast analyses, see Table S4) – none of these were from comparisons between the native populations and the contiguous expansion cluster. Among the successfully annotated outliers, we found 27 genes that were associated with uncharacterised proteins, and 66 genes that were associated with products of potential adaptive importance in invasion (Tables 1; S4). These latter genes can be divided into three main categories: insecticide resistance (e.g., sodium channels/transporters and cadherin), stress tolerance (e.g., heat shock protein and ion transport peptide), and regulation of behaviour (e.g., protein timeless and hormone receptor).

**Table 1:** Key candidate genes located in or within 10 kb of outlier SNPs identified from BayPass genome scan analysis contrasting native versus main invasive clusters of *Bactrocera tryoni*.

Pairwise comparison	Outlier position	Candidate gene	Candidate gene description	Candidate gene function
Native vs. Alice Springs ∩ Native vs. Pacific islands ∩ Native vs. all invasive	NC_052499.1:51371013	LOC120775806	Sodium-coupled monocarboxylate transporter 1	Up-regulated in response to fluralaner insecticide in the common cutworm (Jia et al. 2020); down-regulated at lower temperatures in in the red imported fire ant (Vatanparast et al. 2021)
Native vs. Alice Springs	NC_052499.1:56113393	LOC120771435	Acetylcholinesterase	Insecticide-targeted gene (Jia et al. 2020); its expression was induced under temperature (heat) stress in honey bee workers (Kim et al. 2019)
Native vs. Alice Springs ∩ Native vs. all invasive	NC_052499.1:56530119	LOC120782568	Protein timeless homologue	Production of circadian rhythms including mating behaviour and diapause, involved in adaptation of such processes in response to changes in light and temperature (Tauber et al. 2007)
Native vs. Alice Springs	NC_052499.1:75487809	LOC120782773	AN1-type zinc finger protein 6-like	Important role in growth, aging and responses to biotic and abiotic stresses, e.g. environmental stresses that cause oxidative damage in vivo, such as heat, cold, UV light, and pesticides in Asian honey bee (Guo et al. 2021)
Native vs. Alice Springs ∩ Native vs. Pacific islands	NC_052499.1:75635351	LOC120777560	Cadherin-99C	Cadherin promotes cell adhesion and enhances cellular stability and integrity as a protective mechanism against heat stress in the thermophilic ant genus *Cataglyphis* (Perez et al. 2021); confers resistance to Bt toxin in the cotton bollworm (Gao et al. 2018)
Native vs. Alice Springs ∩ Native vs. Pacific islands	NC_052500.1:18467508	LOC120767202	Trehalose-phosphate phosphatase B	Likely involved in thermotolerance in soldier flies (Stratiomyidae) larvae and stress resistance in other insects (Garbuz et al. 2008)
Native vs. Alice Springs	NC_052501.1:9002087	LOC120770590	Ion transport peptide-like	Encodes thirst-promoting and anti-diuretic hormone in *Drosophila* and involves in its response to osmotic and desiccation stress (Gáliková et al. 2018); encodes neuropeptides in the CNS controlling the evening peak of locomotor activity of the fly (flybase)
Native vs. Alice Springs	NC_052501.1:12026571	LOC120771606	Aquaporin AQPAe.a	Water-selective transmembrane channel that plays a role in desiccation tolerance and maintaining osmotic balance (also in cold hardiness in freeze-tolerant insects) (Chown et al. 2011)
Native vs. Alice Springs	NC_052501.1:17974111	LOC120772702	Sodium channel protein 60E	Involves in membrane excitability and is the target of neurotoxins, including several classes of insecticides (Dong et al. 2014)
Native vs. Alice Springs	NC_052501.1:61156793	LOC120770074	Mucin-19	Mucin mediates cell adhesion and is reported to be downregulated in response to heat stress in the oriental fruit fly (Gu et al. 2019)
Native vs. Alice Springs ∩ Native vs. Pacific islands ∩ Native vs. all invasive	NC_052501.1:86202350	LOC120772156	COP9 signalosome complex subunit 7	Involved in social behaviour, immunity, and adult physiology (Tong et al. 2015) and female fecundity of *Bactrocera dorsalis* (Zhang et al. 2019)
Native vs. Alice Springs	NC_052501.1:86320812	LOC120770414	Hormone receptor 4	Involved in steroid hormone mediated mating behaviour by mediating the timing and expression of steroid hormone 20-hydroxyecdysone to reduce mating propensity in a parasitoid wasp (and other insects) (Ma et al. 2021)
Native vs. Alice Springs ∩ Native vs. Pacific islands	NC_052502.1:49154112	LOC120774945	Odorant receptor 7a-like	Enables detection of hormones, localise food and mates and involved in insecticide susceptibility in some insects (Ha and Smith 2022)
Native vs. Alice Springs	NC_052503.1:20878714	LOC120776680	Solute carrier organic anion transporter family member 74D	Regulates transportation of organic anions in excreting tissues, also, involved in elimination of insecticides in the red flour beetle (Rösner et al. 2021)
Native vs. Alice Springs	NC_052503.1:68462596	LOC120778592	Heat shock 70 kDa protein cognate 1	Critical physiological products under abiotic stress such as thermal stress (Kim et al. 2019; Vatanparast et al. 2021)
Native vs. Alice Springs ∩ Native vs. Pacific islands	NC_052501.1:86078491	LOC120770152	Metabotropic glutamate receptor 2	Functions as a thermal receptor in the peripheral nervous system in *C*. *elegans* (Gong et al. 2019)
Native vs. Pacific islands	NC_052502.1:24557499	LOC120775939	Low-density lipoprotein receptor-related protein 2	Up-regulated in response to fluralaner insecticides in the common cutworm (Jia et al. 2020)

See Table S4 for the full list of outlier SNPs and associated genes.

GO analysis identified significantly enriched pathways (*p* < 0.05) for one molecular function – DNA binding (GO:0003677) - for native vs. all invasive outliers, seven molecular functions – protein binding (GO:0005515), cholinesterase activity (GO:0004104), DNA binding (GO:0003677), metal ion binding (GO:0046872), ATP binding (GO:0005524), zinc ion binding (GO:0008270), and heme binding (GO:0020037) – for native vs. Alice Springs outliers, and two molecular functions – protein binding (GO:0005515) and hydrolase activity (GO:0016787) – for native vs. Pacific Islands outliers. Only one cellular component term, COP9 signalosome (GO:0008180), was significantly enriched for native vs. all invasive outliers as well as Alice Springs and the Pacific Islands outliers.

### Environmental correlates of genomic variation

**Fig. 4:**
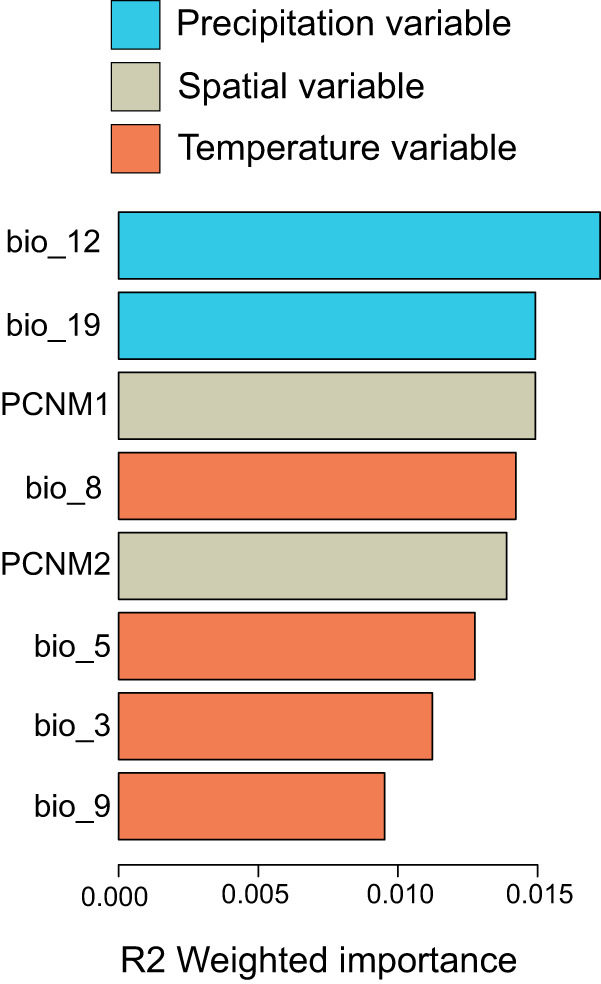
Accuracy importance (R^2^ weighted importance) of the bioclimatic and spatial variables for explaining genetic gradients in *Bactrocera tryoni* according to gradient forest analysis. Bio_12, annual precipitation; bio_19, precipitation of coldest quarter; bio_8, mean temperature of wettest quarter; bio_5, max temperature of warmest month; bio_3, isothermality; bio_9, mean temperature of driest quarter. PCNMs represent axes of spatial patterns.

**Fig. 5:**
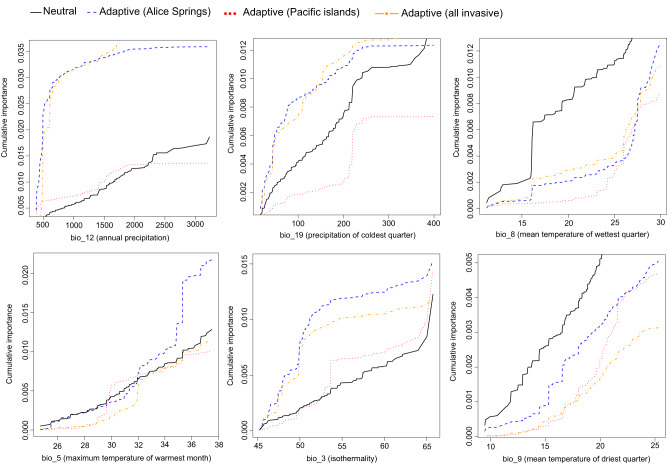
Aggregate compositional turnover functions estimated by gradient forest analysis for the six bioclimate variables of *Bactrocera tryoni*. The pattern of each turnover function shows the response of genetic variation to the bioclimatic gradient. The maximum value of each curve shows the relative importance of the corresponding variable to the change in genetic variation. The BayPass SNP outliers identified for all invasive populations, Alice Springs, and the Pacific Islands were analysed separately from the neutral SNPs.

The GF analysis found that bio_12 (annual precipitation), bio_19 (precipitation of coldest quarter), and bio_8 (mean temperature of the wettest quarter) variables had the highest effect on explaining spatial patterns of allelic turnover (Fig. 4). These results are based on the weighted R^2^ values, which provide a measure of the relative importance of each variable in influencing the observed changes in allele frequencies across the landscape. Spatial location (PCNM1) was another important variable in explaining allelic turnover, showing equal weighted R^2^ value to bio_19 (Fig. 4). Turnover functions for the predictors showed an overall stronger response in the allelic turnover of adaptive SNPs compared to neutral SNPs. This observation was particularly evident when analysing outlier SNPs obtained from contrasting native populations versus all invasive populations and native populations versus Alice Springs in bio_12, bio_19 and bio_3 (Fig. 5). Neutral SNPs showed stronger allelic turnover in bio_5 and bio_9. Markedly, all neutral and adaptive allelic compositions showed a sharp turnover when precipitation (bio_19 and bio_12) and mean temperature (bio_8 and bio_9) approached their minimum values (Fig. 5). All adaptive and neutral SNPs showed relatively similar allelic turnover patterns for bio_5.

## Discussion

We characterised genomic signatures of local adaptation in the invasive Queensland fruit fly, *B. tryoni* across environmentally different habitats in Australia using genome-wide SNP data. We found higher genetic connectivity between native and contiguous introduced populations in southern temperate regions; however, geographically disjunct introduced populations in central Northern Territory and tropical/subtropical Pacific Islands were genetically differentiated. Contrasting allele frequencies of native and introduced populations identified SNPs in close proximity to genes likely to be important in stress tolerance in the geographically and genetically isolated invasive populations. Furthermore, we found precipitation variables, geographic factors, and temperature, to best explain allelic shifts across the current distribution of *B. tryoni*. Our results provide insights into the capacity of this important horticultural pest to adapt to diverse local environments.

### Genetic diversity and connectivity are higher in contiguous expanded range than in disjunct populations

We found varying levels of genetic differentiation and diversity between native and different invasive populations. Invasive populations that contiguously expanded their range down the east coast of Australia into temperate zones showed higher genome-wide admixture and effective migration with the native range compared to disjunct invasive populations. Patterns of isolation by distance increased towards the edges of the contiguous expanded range as well as the geographically disjunct invasive populations. These contiguous expanded populations show lower inbreeding coefficients and marginally higher genomic diversity compared to the disjunct invasive populations. Overall, these SNP patterns corroborate earlier microsatellite studies showing little genetic differentiation between the native and contiguous expanded range, contrary to segregation and a lack of genetic variability in the isolated Alice Springs population (Yu et al. 2001).

Genetically homogenised patterns across wide geographic ranges often evolve in species with high long-distance dispersal capacities. In *B. tryoni*, however, active dispersal rates are relatively low, with mating distance and mean daily dispersal distance predicted to be 16.1 m and 30 m, respectively (Dominiak and Fanson 2023). In this case, trade-facilitated long-distance dispersal, especially during the larval stage inside infested fruit (Meats and Edgerton 2008), might have contributed to the minimal genetic structure of *B. tryoni* seen across the contiguous expanded ranges. Additionally, flies in the southern temperate regions are reported to regularly move short distances between orchards and nearby water sources to compensate for low moisture in the area (Fletcher 1973, 1974; Popa-Báez, Lee et al. 2020). Such short-distance movements could contribute to outbreeding and enhance genetic connectivity across this region.

Fine-scale analysis of the contiguous expanded range revealed that populations at the edge of the contiguous expansion front (Ardlethan, Griffith, and Shepperton) and western expansion front (Cloncurry) were more differentiated. Under the central-marginal hypothesis, higher population differentiation and lower genetic diversity are expected across edge populations relative to core populations due to reduced population sizes and greater isolation and drift (Eckert et al. 2008) and this is supported by our findings of higher isolation and increasingly reduced genetic diversity towards the edges of the contiguous expanded range. Alternatively, serial founder events and allele surfing could also reduce genetic diversity and enhance population differentiation at the expanding edge of an invasion wave (Excoffier and Ray 2008). Such stochastic genetic and population dynamic processes can be especially important for driving rapid genetic differentiation in colonising populations when effective population sizes are small enough for random drift to dominate (Hallatschek et al. 2007; Excoffier and Ray 2008; Trumbo et al. 2016).

### Signatures of local environmental adaptation are stronger in disjunct populations than in contiguous expanded range

Gene flow among populations can enhance or impede local adaptation, depending on the magnitude of genetic connectivity and the strength of local selection pressures (Garant et al. 2007; Sexton et al. 2014). The weak signal of local adaptation and the presence of only two invasion-associated outlier loci in the contiguous expanded range can be attributed to high admixture with native range, potentially swamping locally adapted loci through density-dependent effects (Lenormand 2002; Sexton et al. 2014). High gene flow can stall adaptation to local environments if it occurs among continuous populations along an environmental cline (Sexton et al. 2014). Another explanation for the lack of strong local adaptive signal in much of south-eastern Australia comes from niche modelling, which suggests that moderately to highly suitable habitats for *B*. *tryoni* exist throughout this region (Sultana et al. 2017): local habitat suitability and ecological niche similarity with the native range could have potentially weakened environmental or climatic drive for local adaptation in the contiguous expansion range. However, due to the relatively slow LD decay observed in our dataset (which could have affected capturing candidate genes within our chosen 10 kb distance to outlier SNPs), applying a higher number of SNPs (e.g., low-coverage whole genome resequencing or pool-seq techniques) to this question could potentially elucidate finer-scale patterns of differentiation and local adaptation that are not captured in our studied dataset.

The potential for local adaptation might be highest in small and sparsely populated environments (Lenormand 2002) where reductions in strong gene flow can enhance adaptive differentiation (Sexton et al. 2014). We find stronger genomic evidence for local adaptation (i.e., SNP outliers in genome scans) in Alice Springs and the Pacific Islands, which are geographically disjunct and genetically isolated. These outliers represent population-specific or idiosyncratic genomic signatures that are important for adaptations to their respective environmental conditions, and hence should not indiscriminately be interpreted as adaptive loci directly linked to invasion phenotypes in the broader context of *B. tryoni*. While these outliers provide valuable insights into the capacity of *B. tryoni* to adapt to various environmental conditions, further research is required to investigate adaptive patterns across the entire species range to capture the full complexity of invasion phenotypes in this taxon.

Genes involved in stress resistance were mostly observed in Alice Springs, suggesting that local selection may drive adaptive responses in more arid environments. Among these, heat shock protein, aquaporin and ion transport peptide have well-understood functions in insect adaptation to temperature and moisture stress (Chown et al. 2011; King and MacRae 2015; Gáliková et al. 2018; Perez et al. 2021). Outlier SNPs near insecticide resistance genes included sodium channel protein and cadherin, which are important for the evolution of resistance phenotypes in different insect pests (Dong et al. 2014; Gao et al. 2018). Although insecticide resistance development in Tephritid fruit flies is reported to be slower compared to the other insect pests – mainly due to their high mobility and polyphagy (Vontas et al. 2011) – and no insecticide resistance has been reported for *B. tryoni* to the best our knowledge, our results suggest the potential for the evolution of resistance phenotypes from standing variation. Candidate genes, such as protein timeless and hormone receptor 4, have been implicated in diapause regulation (e.g., in *Drosophila*, Tauber et al. 2007) and mating receptivity in insects (Ma et al. 2021). Additionally, odorant receptors are crucial for insect survival, enabling the detection of environmental cues and vital resources such as food, mates, and predators (Vieira and Rozas 2011).

In addition to these three main groups of candidate genes, we identified SNP outliers in close proximity to several other candidate genes that are involved in development, metabolism, and various cellular and physiological functions. However, due to the limited functional and phenotypic studies on these genes, their putative role in local adaptation and invasion success for insects remains elusive. Future quantitative genetic studies focusing on these genes, as well as the functional effects of the outlier SNPs, will improve understanding of how functional genomic differences across the geographic range of *B*. *tryoni* may facilitate climatic adaptability and/or invasion success.

### Spatial trends in allele frequency turnover

We found that environmental variables and geographic factors play important roles in shaping patterns of allele frequency shifts in *B*. *tryoni*. In particular, precipitation variables showed the highest weighted R^2^ importance in gradient forest model, followed by variables reflecting geography and temperature. These patterns can imply ecological specialty of *B. tryoni* and its irrigation-associated range expansion (Popa-Báez, Catullo et al. 2020). The overall trends in both neutral and adaptive alleles were steep for all the studied bioclimatic variables, confirming the correlative impacts of moisture and temperature in the survival of *B*. *tryoni* across various climatic zones.

An association of genomic and environmental variables can result from geographic, demographic, or selective forces (Wang and Bradburd 2014). Here, it seems unlikely that geographic distance or demography (e.g., population history and structure) alone explain these trends. Notably, several of the loci used in the GF analysis were outlier loci potentially under selection (detected by comparing the native range versus different invasive lineages and the pooled invasive dataset). Some of these loci have well-understood functions related to the tolerance of thermal and desiccation stresses, suggesting the importance of precipitation and temperature in shaping allele frequency trends in the current distribution range of *B. tryoni*. Although non-equilibrium demographic processes can affect the identification of outlier (i.e., putatively adaptive) loci, we reduced these effects by separately comparing each main invasive cluster with the native range in our genome scan, but the limited number of samples available for some of our invasive lineages (such as Alice Springs) could have affected our ability to detect true adaptive signals associated with their invasive status. Also, our outlier detection approach has been shown to be robust to demographic events typical of biological invasions (Olazcuaga et al. 2020). However, our GF analysis may have captured signals of allele frequency changes that originally arose from neutral processes during colonisation and expansion into different invaded areas. These signals may have been further reinforced by distinct environmental conditions that shaped genomic variants in different geographic regions.

## Conclusions

As climate change is driving rapid expansions/shifts in insect ranges globally (Lehmann et al. 2020), understanding how exposure to novel environments can drive adaptive responses among populations is crucial. Genome-wide SNP data allowed us to identify spatial heterogeneity in signatures of local adaptation across populations invading environmentally different ranges in an invasive tropical fruit fly. We found that the degree of connectivity with the native range greatly affected genomic signals of local adaptation in invasive populations. Our findings also supported previous common garden experiments suggesting a genetic basis for complex ecotypic variations without significant association with latitude – in heat, desiccation, and starvation resistance in *B*. *tryoni* (Popa-Báez, Lee et al. 2020). Although a few thousand SNPs used in this study could identify candidate genes important for stress tolerance, applying a higher number of SNPs (e.g., using whole genome resequencing data), could help to uncover finer-scale adaptive patterns as well as explain their underlying molecular evolutionary mechanisms.

### Supplementary information

Supplementary Material

## Data Availability

No new genomic data were generated as part of this study. All genomic data and metadata are available at the Dryad Digital repository (10.5061/dryad.kkwh70s9q) and related scripts are available at GitHub (https://github.com/Elahep/B.tryoni_PopGenomics).
