# Supplementary material for: Genomic signals of local adaptation across climatically heterogenous habitats in an invasive tropical fruit fly (Bactrocera tryoni)
Source: Heredity (Edinb). 2023 Oct 30;132(1):18–29. doi: 10.1038/s41437-023-00657-y (PMC10798995; doi:10.1038/s41437-023-00657-y)
Supplement: Supplementary file 1 — Supplementary Material [file 41437_2023_657_MOESM1_ESM.docx]

**Genomic signals of local adaptation across climatically heterogenous habitats in an invasive tropical fruit fly (*Bactrocera tryoni*)**

Elahe Parvizi^1^, Amy L. Vaughan^2^, Manpreet K. Dhami^2^, Angela McGaughran^1*^

^1^ Te Aka Mātuatua/School of Science, University of Waikato, Hamilton, New Zealand

^2^ Biocontrol and Molecular Ecology, Manaaki Whenua Landcare Research, Lincoln, New Zealand

**Supplementary material**

Contents

[Table S1. 2](#_Toc139887660)

[Table S2 3](#_Toc139887661)

[Table S3 4](#_Toc139887662)

[Table S4 5](#_Toc139887663)

[Figure S1 12](#_Toc139887664)

[Figure S2 13](#_Toc139887665)

[Figure S3 14](#_Toc139887666)

[References 15](#_Toc139887667)

Table S1. Sampling details of *Bactrocera tryoni* for the DarT sequences previously published in Popa-Báez et al. (2020). Locality numbers are used in Figure 1.

| **Population** | **Locality number** | **State** | **Coordinates**  **(latitude, longitude)** | **Historical status** |
| --- | --- | --- | --- | --- |
| Weipa | 1 | Queensland | −12.65, 141.85 | Native |
| Mapoon | 2 | Queensland | −11.87, 142.19 | Native |
| Cape York | 3 | Queensland | −10.70, 142.51 | Native |
| Coen | 4 | Queensland | −13.94, 143.20 | Native |
| Cooktown | 5 | Queensland | −15.48, 145.25 | Native |
| Cape Tribulation | 6 | Queensland | −16.09, 145.46 | Native |
| Mareeba | 7 | Queensland | −17.00, 145.44 | Native |
| Cairns | 8 | Queensland | −16.89, 145.74 | Native |
| Utchee Creek | 9 | Queensland | −17.63, 145.92 | Native |
| Townsville | 10 | Queensland | −19.28, 146.80 | Native |
| Bowen | 11 | Queensland | −20.02, 148.22 | Native |
| Rockhampton | 12 | Queensland | −23.38, 150.51 | Native |
| Brisbane | 13 | Queensland | −27.41, 152.94 | Native |
| Torrens Creek | 14 | Queensland | −20.77, 145.02 | Native |
| Hughenden | 15 | Queensland | −20.85, 144.20 | Native |
| Cloncurry | 16 | Queensland | −20.71, 140.51 | Invasive |
| Mt Isa | 17 | Queensland | −20.73, 139.49 | Invasive |
| Narrabri | 18 | New South Wales | −30.33, 149.78 | Invasive |
| Sydney | 19 | New South Wales | −33.87, 151.21 | Invasive |
| Canberra | 20 | New South Wales | −35.40, 149.10 | Invasive |
| Batemans Bay | 21 | New South Wales | −35.71, 150.18 | Invasive |
| Bega Valley | 22 | New South Wales | −36.62, 149.97 | Invasive |
| Ardlethan | 23 | New South Wales | −34.35, 146.90 | Invasive |
| Griffith | 24 | New South Wales | −34.28, 146.05 | Invasive |
| Shepparton | 25 | Victoria | −36.38, 145.40 | Invasive |
| Alice Springs | 26 | Northern Territory | −23.70, 133.88 | Invasive |
| Loyalty Island | 27 | Pacific Island | −21.01, 167.22 | Invasive |
| Tahiti | 28 | Pacific Island | −17.56, −149.56 | Invasive |

Table S2. Estimates of population genetic indices based on 6,707 SNPs in native and introduced ranges of *Bactrocera tryoni*. Locality numbers are used in Figure 1. Native populations are highlighted in yellow and introduced populations are highlighted in blue. n: number of individuals; HO: average observed heterozygosity; HE: average expected heterozygosity; π: average nucleotide diversity; F_IS_: inbreeding coefficient; S.E.: standard error.

| Population | Locality number | n | HO | S.E. | HE | S.E. | π | S.E. | F_IS_ | S.E. |
| --- | --- | --- | --- | --- | --- | --- | --- | --- | --- | --- |
| Weipa | 1 | 5 | 0.25392 | 0.00271 | 0.20265 | 0.0019 | 0.22575 | 0.00212 | -0.06331 | 0.00375 |
| Mapoon | 2 | 8 | 0.25597 | 0.00241 | 0.20953 | 0.00171 | 0.2238 | 0.00183 | -0.08431 | 0.00492 |
| Cape York | 3 | 11 | 0.25493 | 0.00217 | 0.21202 | 0.00158 | 0.22247 | 0.00166 | -0.09414 | 0.00715 |
| Coen | 4 | 8 | 0.25141 | 0.00243 | 0.20517 | 0.00173 | 0.21992 | 0.00185 | -0.08137 | 0.00747 |
| Cooktown | 5 | 8 | 0.25429 | 0.00234 | 0.20839 | 0.00167 | 0.22252 | 0.00178 | -0.08474 | 0.00444 |
| Cape Tribulation | 6 | 7 | 0.25686 | 0.00252 | 0.20425 | 0.00175 | 0.2206 | 0.00189 | -0.09057 | 0.00584 |
| Mareeba | 7 | 4 | 0.25198 | 0.00308 | 0.19258 | 0.00209 | 0.22497 | 0.00246 | -0.05284 | 0.00661 |
| Cairns | 8 | 21 | 0.25195 | 0.00191 | 0.21029 | 0.00137 | 0.21564 | 0.0014 | -0.11912 | 0.01466 |
| Utchee Creek | 9 | 9 | 0.25777 | 0.00237 | 0.20807 | 0.00165 | 0.22062 | 0.00175 | -0.0998 | 0.00561 |
| Townsville | 10 | 8 | 0.25192 | 0.00237 | 0.20627 | 0.00169 | 0.22027 | 0.0018 | -0.08396 | 0.00459 |
| Bowen | 11 | 8 | 0.25638 | 0.00238 | 0.20979 | 0.0017 | 0.22408 | 0.00181 | -0.08544 | 0.0048 |
| Rockhampton | 12 | 12 | 0.25182 | 0.00215 | 0.20669 | 0.00152 | 0.21624 | 0.00159 | -0.10432 | 0.01272 |
| Brisbane | 13 | 22 | 0.25165 | 0.0019 | 0.21036 | 0.00137 | 0.21552 | 0.0014 | -0.1192 | 0.01783 |
| Torrens Creek | 14 | 8 | 0.25622 | 0.00235 | 0.20766 | 0.00166 | 0.22191 | 0.00177 | -0.09102 | 0.00561 |
| Hughenden | 15 | 8 | 0.25503 | 0.00238 | 0.20628 | 0.00166 | 0.22037 | 0.00178 | -0.09096 | 0.00495 |
| Cloncurry | 16 | 8 | 0.2537 | 0.00278 | 0.19851 | 0.00188 | 0.21209 | 0.00201 | -0.10135 | 0.00584 |
| Mt Isa | 17 | 8 | 0.25253 | 0.00259 | 0.20094 | 0.00179 | 0.21468 | 0.00191 | -0.09583 | 0.00556 |
| Narrabri | 18 | 9 | 0.25556 | 0.00233 | 0.20725 | 0.00163 | 0.21974 | 0.00173 | -0.09721 | 0.00534 |
| Sydney | 19 | 15 | 0.25235 | 0.00209 | 0.20825 | 0.00149 | 0.21595 | 0.00155 | -0.11108 | 0.01328 |
| Canberra | 20 | 16 | 0.25506 | 0.00207 | 0.21072 | 0.00148 | 0.21774 | 0.00153 | -0.11538 | 0.00969 |
| Batemans Bay | 21 | 9 | 0.25004 | 0.00236 | 0.20296 | 0.00167 | 0.21525 | 0.00177 | -0.09444 | 0.00631 |
| Bega Valley | 22 | 13 | 0.25037 | 0.00225 | 0.20472 | 0.0016 | 0.21329 | 0.00166 | -0.10752 | 0.01012 |
| Ardlethan | 23 | 8 | 0.25773 | 0.00253 | 0.20586 | 0.00175 | 0.2199 | 0.00187 | -0.09688 | 0.00497 |
| Griffith | 24 | 10 | 0.25296 | 0.00249 | 0.20281 | 0.00172 | 0.21379 | 0.00182 | -0.10434 | 0.00681 |
| Shepparton | 25 | 9 | 0.25247 | 0.00246 | 0.20291 | 0.00171 | 0.21517 | 0.00181 | -0.09847 | 0.00598 |
| Alice Springs | 26 | 31 | 0.25626 | 0.00262 | 0.20261 | 0.00179 | 0.20606 | 0.00182 | -0.14266 | 0.023 |
| Loyalty Island | 27 | 10 | 0.25758 | 0.00293 | 0.1986 | 0.00196 | 0.20963 | 0.00207 | -0.11667 | 0.00953 |
| Tahiti | 28 | 8 | 0.25653 | 0.0035 | 0.1861 | 0.00221 | 0.19929 | 0.00238 | -0.12388 | 0.00991 |

Table S3. Pairwise population F_ST_ values showing genetic distances between native and introduced populations of *Bactrocera tryoni* based on 6,707 SNPs. Red colour represents lower genetic differentiation while green shows higher population isolation.

Table S4. Full list of candidate genes located at close proximity (10 kb) to BayPass outlier SNPs identified by comparing native populations versus all invasive populations, as well as native populations versus southern expanded ranges, Alice Springs, and the Pacific Islands in *Bactrocera tryoni*. Outliers highlighted in blue are common between different contrast analyses.

| **Pairwise comparison** | **SNP outlier position** | **Candidate gene ID** | **Candidate gene position** | **Candidate gene description** | **Candidate gene function (from studies on *Drosophila* or other species)** | **Reference** |
| --- | --- | --- | --- | --- | --- | --- |
| Native vs. all invasive | NC_052499.1:9538889 | no exons | NA | NA | NA |  |
| Native vs. all invasive | NC_052499.1:43308649 | LOC120771228 | NC_052499.1 (43050120..43464343) | mediator of RNA polymerase II transcription subunit 15-like | controls transcription from class II genes; development & reproduction | (Blazek et al., 2005); flybase |
| Native vs. all invasive | NC_052499.1:51371013 | LOC120775806 | NC_052499.1 (51376881..51526588) | sodium-coupled monocarboxylate transporter 1 | up-regulated in response to fluralaner insecticide in the common cutworm *Spodoptera litura*; down-regulated at lower temperatures in in the red imported fire ant (*Solenopsis invicta*) | (Jia et al., 2020; Vatanparast et al., 2021) |
| Native vs. all invasive | NC_052499.1:56530119 | LOC120782568 | NC_052499.1 (56224854..56720530) | protein timeless homolog | production of circadian rhythms including mating behaviour and diapause, involved in adaptation of such processes in response to changes in light and temperature | (Tauber et al., 2007) |
| Native vs. all invasive | NC_052500.1:17036657 | LOC120769096 | NC_052500.1 (17022685..17069149 | homeotic protein spalt-major | Required for the establishment of the posterior-most head and the anterior-most tail segments of the embryo in *Drosophila*. | flybase |
| Native vs. all invasive | NC_052500.1:76636749 | LOC120769762 | NC_052500.1 (76539399..76724385) | protein FAM102B | involved in dsRNA transport | flybase |
| Native vs. all invasive | NC_052501.1:83441072 | no exons | NA | NA | NA |  |
| Native vs. all invasive | NC_052501.1:86202350 | LOC120772156 | NC_052501.1 (86173787..86197464) | COP9 signalosome complex subunit 7 | involved in social behaviour, immunity, and adult physiology | (Tong et al., 2015; Zhang et al., 2019) |
| Native vs. all invasive | NC_052502.1:36640935 | LOC120774554 | NC_052502.1 (36636794..36651833) | ubiquitin-conjugating enzyme E2 H | involved in protein ubiquitination, a process which is essential for recovery of cellular activities after heat shock (in vitro experiment) | Flybase; (Maxwell et al., 2021) |
| Native vs. all invasive | NC_052502.1:40677125 | LOC120775878 | NC_052502.1 (40665184..40677161) | extensin-2-like | NA |  |
| Native vs. all invasive | NC_052502.1:53034145 | LOC120774360 | NC_052502.1 (53042562..53047202) | uncharacterized | NA |  |
| Native vs. all invasive | NC_052502.1:68285560 | LOC120774364 | NC_052502.1 (68074338..68289322) | homeobox protein cut | regulates cell proliferation and patterning | (Pitsouli & Perrimon, 2013) |
| Native vs. all invasive | NC_052502.1:71987739 | no exons | NA | NA | NA |  |
| Native vs. all invasive | NC_052503.1:22794084 | no exons | NA | NA | NA |  |
| Native vs. all invasive | NC_052503.1:31352727 | no exons | NA | NA | NA |  |
| Native vs. all invasive | NW_024395342.1:8297 | no exons | NA | NA | NA |  |
| Native vs. southern expanded | NC_052503.1:31352727 | no exons | NA | NA | NA |  |
| Native vs. southern expanded | NW_024395342.1:8297 | no exons | NA | NA | NA |  |
| Native vs. Alice Springs | NC_052499.1:27573071 | LOC120766489 LOC120782476 | NC_052499.1 (27491592..27630121) NC_052499.1 (27556610..27580906) | ras-like protein family member 10B odorant receptor (OR) coreceptor | development, cell proliferation and differentiation localization of ORs to dendritic membranes and odorant detection | (Ogura et al., 2009; Stengl & Funk, 2013) |
| Native vs. Alice Springs | NC_052499.1:43308649 | LOC120771228 | NC_052499.1 (43050120..43464343) | mediator of RNA polymerase II transcription subunit 15-like | controls transcription from class II genes; development & reproduction | (Blazek et al., 2005); flybase |
| Native vs. Alice Springs | NC_052499.1:48889636 | no exons | NA | NA | NA |  |
| Native vs. Alice Springs | NC_052499.1:50494563 | no exons | NA | NA | NA |  |
| Native vs. Alice Springs | NC_052499.1:51371013 | LOC120775806 | NC_052499.1 (51376881..51526588) | sodium-coupled monocarboxylate transporter 1 | up-regulated in response to fluralaner insecticide in the common cutworm *Spodoptera litura*; down-regulated at lower temperatures in in the red imported fire ant (*Solenopsis invicta*) | (Jia et al., 2020; Vatanparast et al., 2021) |
| Native vs. Alice Springs | NC_052499.1:53821686 | no exons | NA | NA | NA |  |
| Native vs. Alice Springs | NC_052499.1:56113393 | LOC120771435 | NC_052499.1 (55959550..56153912) | acetylcholinesterase | insecticide-targeted gene; its expression was induced under temperature (heat) stress in honey bee workers | (Jia et al., 2020; Kim et al., 2019) |
| Native vs. Alice Springs | NC_052499.1:56530119 | LOC120782568 | NC_052499.1 (56224854..56720530) | protein timeless homolog | production of circadian rhythms including mating behaviour and diapause, involved in adaptation of such processes in response to changes in light and temperature | (Tauber et al., 2007) |
| Native vs. Alice Springs | NC_052499.1:61062936 | LOC120766280 | NC_052499.1 (61039057..61116531) | pituitary homeobox homolog Ptx1 | control physiological cell functions during development | flybase |
| Native vs. Alice Springs | NC_052499.1:62164506 | LOC120766782 | NC_052499.1 (62048211..62368441) | homeobox protein homothorax | regulates expression of photoreceptors as light-sensing structures; involves in development, proximal appendages patterning, and cell division promotion | (Mishra et al., 2021); flybase |
| Native vs. Alice Springs | NC_052499.1:62547367 | no exons | NA | NA | NA |  |
| Native vs. Alice Springs | NC_052499.1:65597876 | no exons | NA | NA | NA |  |
| Native vs. Alice Springs | NC_052499.1:67491876 | LOC120774377 | NC_052499.1 (67493283..67494029) | uncharacterized | NA |  |
| Native vs. Alice Springs | NC_052499.1:67796025 | LOC120768537 | NC_052499.1 (67672038..67809873) | uncharacterized | NA |  |
| Native vs. Alice Springs | NC_052499.1:74549310 | LOC120776053 | NC_052499.1 (74382668..74586475) | hemicentin-1 | cell adhesion; involved in adaptation to thermal stress in corals | (Kirk et al., 2018; Vargas et al., 2022) |
| Native vs. Alice Springs | NC_052499.1:75487809 | LOC120782773 | NC_052499.1 (75461797..75482598) | AN1-type zinc finger protein 6-like | important role in growth, aging and responses to biotic and abiotic stresses (e.g. environmental stresses that cause oxidative damage in vivo, such as heat, cold, UV light, and pesticides in *Apis cerana*) | (Guo et al., 2021) |
| Native vs. Alice Springs | NC_052499.1:75635351 | LOC120777560 | NC_052499.1 (75570372..75641059) | cadherin-99C | promotes cell adhesion and enhances cellular stability and integrity as a protective mechanism against heat stress in the thermophilic ant genus *Cataglyphis*; confers resistance to Bt toxin in transgenic cotton | (Gao et al., 2018; Perez et al., 2021) |
| Native vs. Alice Springs | NC_052499.1:76576632 | LOC120777855 | NC_052499.1 (76404074..76627390) | uncharacterized | NA |  |
| Native vs. Alice Springs | NC_052499.1:76831959 | LOC120779132 | NC_052499.1 (76671311..76872645) | uncharacterized | NA |  |
| Native vs. Alice Springs | NC_052499.1:80769784 | no exons | NA | NA | NA |  |
| Native vs. Alice Springs | NC_052499.1:85346290 | LOC120766738 LOC120766736 LOC120779175 | NC_052499.1 (85330178..85358999) NC_052499.1 (85334294..85547645) NC_052499.1 (85341260..85352664) | caspase-3-like collagen alpha chain CG42342 uncharacterized | important role in apoptosis and inflammation, associated with immune system response to salinity stress in mosquitoes; muscle structure and function (e.g. insect flight activity) | (Jones et al., 2015; Uyhelji et al., 2016) |
| Native vs. Alice Springs | NC_052499.1:87473054 | LOC120774297 | NC_052499.1 (87398130..87516097) | uncharacterized | NA |  |
| Native vs. Alice Springs | NC_052499.1:87473514 | LOC120774297 | NC_052499.1 (87398130..87516097) | uncharacterized | NA |  |
| Native vs. Alice Springs | NC_052499.1:88812046 | LOC120767072 | NC_052499.1 (88758331..88864521) | guanylate cyclase soluble subunit beta-1 | involves in neuronal development (e.g. photoreceptors) and response to hypoxia | (Morton & Vermehren, 2007) |
| Native vs. Alice Springs | NC_052499.1:89170604 | no exons | NA | NA | NA |  |
| Native vs. Alice Springs | NC_052500.1:977719 | LOC120767682 | NC_052500.1 (956681..985312) | probable ATP-dependent permease | transports various molecules including bioamines, neurotransmitters and metabolic intermediates | flybase |
| Native vs. Alice Springs | NC_052500.1:16608169 | no exons | NA | NA | NA |  |
| Native vs. Alice Springs | NC_052500.1:18467508 | LOC120768270 LOC120767202 | NC_052500.1 (18456542..18457851) NC_052500.1 (18459100..18475526) | protein YIPF6 trehalose-phosphate phosphatase B | transmembrane protein located in Golgi apparatus and ER likely involved in thermotolerance in soldier flies (Stratiomyidae) larvae | flybase (Garbuz et al., 2008) |
| Native vs. Alice Springs | NC_052500.1:21050807 | LOC120767328 | NC_052500.1 (21048044..21116411) | uncharacterized | NA |  |
| Native vs. Alice Springs | NC_052500.1:34298632 | no exons | NA | NA | NA |  |
| Native vs. Alice Springs | NC_052500.1:46309826 | no exons | NA | NA | NA |  |
| Native vs. Alice Springs | NC_052500.1:51137017 | LOC120769259 | NC_052500.1 (51135788..51303832) | protein outspread | involved in cell organization and cytoskeletal binding | flybase |
| Native vs. Alice Springs | NC_052500.1:52006698 | no exons | NA | NA | NA |  |
| Native vs. Alice Springs | NC_052500.1:60188524 | no exons | NA | NA | NA |  |
| Native vs. Alice Springs | NC_052500.1:74805999 | no exons | NA | NA | NA |  |
| Native vs. Alice Springs | NC_052500.1:76636749 | LOC120769762 | NC_052500.1 (76539399..76724385) | protein FAM102B | involved in dsRNA transport | flybase |
| Native vs. Alice Springs | NC_052500.1:78091361 | LOC120769090 LOC120769120 | NC_052500.1 (78015222..78088808) NC_052500.1 (78096485..78097837) | protein obstructor-E uncharacterized | chitin-binding protein important for expandability of the larval cuticle (maybe a better protect for body under harsh environmental conditions), also, involved in carbohydrate metabolism | flybase, (von Wyschetzki et al., 2015) |
| Native vs. Alice Springs | NC_052501.1:2626717 | no exons | NA | NA | NA |  |
| Native vs. Alice Springs | NC_052501.1:7386215 | LOC120773042 | NC_052501.1 (7037466..7401997) | uncharacterized | NA |  |
| Native vs. Alice Springs | NC_052501.1:9002087 | LOC120770590 | NC_052501.1 (8979288..9080363) | ion transport peptide-like | encodes thirst-promoting and anti-diuretic hormone in *Drosophila* and involves in response to osmotic and desiccation stress in *Drosophila,* also, encodes neuropeptides in the CNS controlling the evening peak of locomotor activity of the fly | (Gáliková et al., 2018), flybase |
| Native vs. Alice Springs | NC_052501.1:10419921 | LOC120772869 | NC_052501.1 (10368266..10482459) | uncharacterized | NA |  |
| Native vs. Alice Springs | NC_052501.1:12026571 | LOC120771606 | NC_052501.1 (12034612..12055362) | aquaporin AQPAe.a | water-selective transmemberane channel that plays a role in desiccation and maintaining osmotic balance (also in cold hardiness in freeze-tolerant insects) | (Chown et al., 2011) |
| Native vs. Alice Springs | NC_052501.1:14700025 | LOC120771906 | NC_052501.1 (14690247..14693654) | endoplasmic reticulum junction formation protein lunapark-A | involved in network formation and organization of endoplasmic reticulum | flybase |
| Native vs. Alice Springs | NC_052501.1:14990223 | LOC120770991 | NC_052501.1 (14819287..15098585) | protein Shroom | mediates cell organization and biogenesis and embryonic morphogenesis | flybase |
| Native vs. Alice Springs | NC_052501.1:15106302 | LOC120770991 | NC_052501.1 (14819287..15098585) | protein Shroom | mediates cell organization and biogenesis and embryonic morphogenesis | flybase |
| Native vs. Alice Springs | NC_052501.1:17974111 | LOC120772702 | NC_052501.1 (17974011..18258836) | sodium channel protein 60E | involves in membrane excitability and is the target of neurotoxins, including several classes of insecticides | (Dong, 2007) |
| Native vs. Alice Springs | NC_052501.1:61156793 | LOC120770074 | NC_052501.1 (61098675..61485710) | mucin-19 | reported to be downregulated in response to heat stress in *Bactrocera dorsalis,* also, cell adhesion | (Gu et al., 2019), flybase |
| Native vs. Alice Springs | NC_052501.1:66038002 | no exons | NA | NA | NA |  |
| Native vs. Alice Springs | NC_052501.1:74657842 | no exons | NA | NA | NA |  |
| Native vs. Alice Springs | NC_052501.1:83441072 | no exons | NA | NA | NA |  |
| Native vs. Alice Springs | NC_052501.1:83731408 | no exons | NA | NA | NA |  |
| Native vs. Alice Springs | NC_052501.1:84431403 | no exons | NA | NA | NA |  |
| Native vs. Alice Springs | NC_052501.1:85634730 | LOC120770802 LOC120770528 | NC_052501.1 (85615112..85636192) NC_052501.1 (85643322..85644766) | pro-resilin uncharacterized | provides elasticity and involved in storing energy for jumping or flying of insects (specifically, involved in elastic energy conversion with high efficiency during mechanical stress in *Drosophila*) | (Qin et al., 2012) |
| Native vs. Alice Springs | NC_052501.1:86078491 | LOC120770152 LOC120770153 | NC_052501.1 (86040271..86081443) NC_052501.1 (86081444..86083781) | metabotropic glutamate receptor 2 maltase A1-like | involved in cold sensing behaviour (in *C. elegans*) mediates hydrolysis and carbohydrate metabolism, potentially involved in long-distance flight (was upregulated in migratory forms of hoverflies) | (Gong et al., 2019) (Doyle et al., 2022) |
| Native vs. Alice Springs | NC_052501.1:86202350 | LOC120772156 | NC_052501.1 (86173787..86197464) | COP9 signalosome complex subunit 7 | involved in social behaviour, immunity, and adult physiology | (Tong et al., 2015; Zhang et al., 2019) |
| Native vs. Alice Springs | NC_052501.1:86320812 | LOC120770414 | NC_052501.1 (86261172..86415449) | hormone receptor 4 | involved in steroid hormone mediated mating behaviour by mediating the timing and expression of steroid hormone 20-hydroxyecdysone during the onset of metamorphosis | (Ma et al., 2021) |
| Native vs. Alice Springs | NC_052501.1:86940797 | LOC120772107 LOC120770816 | NC_052501.1 (86905296..86933624) NC_052501.1 (86939701..86941868) | neuropeptide CCHamide-2 receptor putative gustatory receptor 36b | mediates feeding behaviour and an important factor for controlling developmental timing in *Drosophila,* also, reported to be downregulated under temperature stresses in Silverleaf whitefly *Bemisia tabaci* chemosensory receptor mediating acceptance or avoidance behaviour (behavioural avoidance has been suggested to be associated with insecticide resistance, see Crossley et al., 2017) | (Li et al., 2021; Ren et al., 2015); flybase |
| Native vs. Alice Springs | NC_052502.1:5273 | LOC120773539 | NC_052502.1 (502..91446) | plectin | involved in cytoskeleton organization and dynamics, also, controls osmoregulation in fish | (Dennenmoser et al., 2017; Wiche, 1998) |
| Native vs. Alice Springs | NC_052502.1:45723 | LOC120773539 | NC_052502.1 (502..91446) | plectin | involved in cytoskeleton organization and dynamics, also, controls osmoregulation in fish | (Dennenmoser et al., 2017; Wiche, 1998) |
| Native vs. Alice Springs | NC_052502.1:2150024 | LOC120774292 | NC_052502.1 (2128021..2158814) | uncharacterized | NA |  |
| Native vs. Alice Springs | NC_052502.1:4482247 | no exons | NA | NA | NA |  |
| Native vs. Alice Springs | NC_052502.1:5231348 | LOC120773595 LOC120775219 LOC120773392 | NC_052502.1 (5221518..5226538) NC_052502.1 (5230292..5231559) NC_052502.1 (5235187..5297385) | uncharacterized E3 ubiquitin-protein ligase MARCHF7 titin | NA involved in protein turnover and removal (e.g. under thermal stress in corals) muscle specific protein involved in insect locomotion, also, interacts with heat shock proteins to protect myofibrils under stress (e.g. heat) conditions in fish | (Mayfield et al., 2014; Tucker & Shelden, 2009) |
| Native vs. Alice Springs | NC_052502.1:5547405 | no exons | NA | NA | NA |  |
| Native vs. Alice Springs | NC_052502.1:5797246 | LOC120774319 | NC_052502.1 (5779357..5826519) | uncharacterized | NA |  |
| Native vs. Alice Springs | NC_052502.1:6588121 | no exons | NA | NA | NA |  |
| Native vs. Alice Springs | NC_052502.1:6612417 | LOC120775870 | NC_052502.1 (6615051..6677061) | uncharacterized | NA |  |
| Native vs. Alice Springs | NC_052502.1:6687972 | no exons | NA | NA | NA |  |
| Native vs. Alice Springs | NC_052502.1:9468688 | LOC120773614 | NC_052502.1 (9478012..9493497) | insulin-like growth factor-binding protein complex acid labile subunit | possible role in immunity and development | flybase |
| Native vs. Alice Springs | NC_052502.1:9569629 | LOC120775626 LOC120774218 LOC120774217 | NC_052502.1 (9572288..9574494) NC_052502.1 (9574673..9576827) NC_052502.1 (9576818..9580157) | N-acetylglucosamine-6-phosphate deacetylase uncharacterized protein F54F2.9  anaphase-promoting complex subunit 4 | involved in catabolic processes related to N-acetylglucosamine regulates mitotic metaphase/anaphase transition, also, has been reported to actively participates in the heat shock response in vitro | flybase; (Ahlskog et al., 2010) |
| Native vs. Alice Springs | NC_052502.1:13304475 | no exons | NA | NA | NA |  |
| Native vs. Alice Springs | NC_052502.1:16397273 | LOC120775337 | NC_052502.1 (16161423..16538299) | platelet binding protein GspB | mediates the binding of bacteria and human platelets | UniProt |
| Native vs. Alice Springs | NC_052502.1:20248230 | LOC120776055 | NC_052502.1 (20190519..20291517) | ras-related protein Rap-2a | enables GTP binding and GTPase activity, also, ras proteins are involved in development, cell proliferation and differentiation | Flybase; (Ogura et al., 2009) |
| Native vs. Alice Springs | NC_052502.1:21787575 | LOC120776065 LOC120774860 | NC_052502.1 (21678844..21784807) NC_052502.1 (21795139..21964793) | uncharacterized uncharacterized | NA |  |
| Native vs. Alice Springs | NC_052502.1:22000285 | no exons | NA | NA | NA |  |
| Native vs. Alice Springs | NC_052502.1:36640935 | LOC120774554 | NC_052502.1 (36636794..36651833) | ubiquitin-conjugating enzyme E2 H | involved in protein ubiquitination, a process which is essential for recovery of cellular activities after heat shock (in vitro experiment) | Flybase; (Maxwell et al., 2021) |
| Native vs. Alice Springs | NC_052502.1:49154112 | LOC120774945 | NC_052502.1 (49157048..49158468) | odorant receptor 7a-like | enables detection of volatile chemicals | flybase |
| Native vs. Alice Springs | NC_052502.1:53034145 | LOC120774360 | NC_052502.1 (53042562..53047202) | uncharacterized | NA |  |
| Native vs. Alice Springs | NC_052502.1:67915209 | no exons | NA | NA | NA |  |
| Native vs. Alice Springs | NC_052502.1:68285560 | LOC120774364 | NC_052502.1 (68074338..68289322) | homeobox protein cut | regulates cell proliferation and patterning | (Pitsouli & Perrimon, 2013) |
| Native vs. Alice Springs | NC_052502.1:75107408 | LOC120774195 | NC_052502.1 (75065188..75142600) | calcitonin gene-related peptide type 1 receptor | regulates calcium homeostasis and osmoregulatory adaptation in fish | (Garcia-Elfring et al., 2021; Kusakabe et al., 2017) |
| Native vs. Alice Springs | NC_052502.1:77675855 | LOC120773626 LOC120773448 | NC_052502.1 (77640416..77670049) NC_052502.1 (77673548..77706381) | carboxypeptidase D arginase-1 | mediates neuropeptide processing important for locomotor activity and survival rates in *Drosophila* involved in development and insect flight metabolism | (Pauls et al., 2019; Raghupathi Reddy & Campbell, 1969) |
| Native vs. Alice Springs | NC_052503.1:8653278 | LOC120778742 | NC_052503.1 (8640807..8645435) | uncharacterized | NA |  |
| Native vs. Alice Springs | NC_052503.1:13126971 | LOC120776683 | NC_052503.1 (13113127..13215810) | putative uncharacterized protein DDB_G0282129 | NA |  |
| Native vs. Alice Springs | NC_052503.1:15918209 | LOC120779190 | NC_052503.1 (15913262..15951995) | myb-like protein Q | enables DNA binding and is involved in regulation of cell cycle. In plants, some members of MYB protein family are involved in regulation of cell death and stress tolerance. | Flybase; (De Vos et al., 2006) |
| Native vs. Alice Springs | NC_052503.1:20878714 | LOC120776680 | NC_052503.1 (20886753..20961256) | solute carrier organic anion transporter family member 74D | regulates transportation of organic anions in excreting tissues, also, involved in elimination of insecticides (e.g., in the red flour beetle, *Tribolium castaneum*) | (Rösner et al., 2021) |
| Native vs. Alice Springs | NC_052503.1:22794084 | no exons | NA | NA | NA |  |
| Native vs. Alice Springs | NC_052503.1:24153057 | no exons | NA | NA | NA |  |
| Native vs. Alice Springs | NC_052503.1:31352727 | no exons | NA | NA | NA |  |
| Native vs. Alice Springs | NC_052503.1:32164053 | no exons | NA | NA | NA |  |
| Native vs. Alice Springs | NC_052503.1:38617279 | no exons | NA | NA | NA |  |
| Native vs. Alice Springs | NC_052503.1:39926419 | LOC120778234 LOC120778236 | NC_052503.1 (39784682..39943102) NC_052503.1 (39913751..39926853) | serine/threonine-protein kinase pakD putative gustatory receptor 77a | involved in regulating cytoskeleton, cell organization and development chemosensory receptor mediating acceptance or avoidance behaviour (behavioural avoidance has been suggested to be associated with insecticide resistance, see Crossley et al., 2017) | (Garcia et al., 2014); flybase |
| Native vs. Alice Springs | NC_052503.1:40252145 | LOC120776567 | NC_052503.1 (39976327..40264030) | cyclin-dependent kinase 14 | involved in the regulation of cell cycle | flybase |
| Native vs. Alice Springs | NC_052503.1:63027269 | LOC120777402 | NC_052503.1 (62806204..63041737) | uncharacterized | NA |  |
| Native vs. Alice Springs | NC_052503.1:68462596 | LOC120776608 LOC120778592 | NC_052503.1 (68436687..68456979) NC_052503.1 (68459482..68465792) | SRSF protein kinase 1  heat shock 70 kDa protein cognate 1 | enables protein serine/threonine kinase activity and involves in mRNA splicing regulation critical physiological products under abiotic stress (heat, cold, crowding, and anoxia) & up-regulated by cold and heat stress (hsp is considered as a general stress marker) | flubase; (Bustos et al., 2020; Kim et al., 2019; King & MacRae, 2015; Vatanparast et al., 2021) |
| Native vs. Alice Springs | NC_052503.1:69431136 | LOC120778228 LOC120777567 LOC120777565 | NC_052503.1 (69420293..69425331) NC_052503.1 (69432619..69433712) NC_052503.1 (69437200..69438850) | uncharacterized  uncharacterized  transcription factor Adf-1-like | NA NA regulates the expression of Adh (alcohol dehydrogenase) and other genes | flybase |
| Native vs. Alice Springs | NC_052503.1:69720163 | LOC120777228 | NC_052503.1 (69445489..69778709) | mucin-17 | reported to be downregulated in response to heat stress in the oriental fruit fly *Bactrocera dorsalis,* also, cell adhesion | (Gu et al., 2019); flybase |
| Native vs. Alice Springs | NC_052503.1:75305442 | no exons | NA | NA | NA |  |
| Native vs. Alice Springs | NC_052503.1:75329191 | LOC120776669 | NC_052503.1 (75319124..75323038) | uncharacterized | NA |  |
| Native vs. Alice Springs | NW_024395342.1:8297 | no exons | NA | NA | NA |  |
| Native vs. Alice Springs | NW_024395977.1:21674826 | LOC120780798 LOC120780339 | NW_024395977.1 (21644681..21690549) NW_024395977.1 (21668945..21669460) | protein unc-13 homolog 4B uncharacterized | involved in synaptic vesicle exocytosis NA | flybase |
| Native vs. Alice Springs | NW_024396193.1:81243 | no exons | NA | NA | NA |  |
| Native vs. Alice Springs | NW_024396313.1:109657 | LOC120781358 | NW_024396313.1 (20527..117811) | discoidin domain-containing receptor 2-like | enables collagen binding | flybase |
| Native vs. Alice Springs | NW_024396573.1:540274 | no exons | NA | NA | NA |  |
| Native vs. Pacific Islands | NC_052499.1:43308649 | LOC120771228 | NC_052499.1 (43050120..43464343) | mediator of RNA polymerase II transcription subunit 15-like | controls transcription from class II genes; development & reproduction | (Blazek et al., 2005); flybase |
| Native vs. Pacific Islands | NC_052499.1:50494563 | no exons | NA | NA | NA |  |
| Native vs. Pacific Islands | NC_052499.1:51371013 | LOC120775806 | NC_052499.1 (51376881..51526588) | sodium-coupled monocarboxylate transporter 1 | up-regulated in response to fluralaner insecticide in the common cutworm *Spodoptera litura*; down-regulated at lower temperatures in in the red imported fire ant (*Solenopsis invicta*) | (Jia et al., 2020; Vatanparast et al., 2021) |
| Native vs. Pacific Islands | NC_052499.1:59142301 | LOC120773468 | NC_052499.1 (59097281..59161930) | mucin-2-like | reported to be downregulated in response to heat stress in *Bactrocera dorsalis,* also, cell adhesion | (Gu et al., 2019); flybase |
| Native vs. Pacific Islands | NC_052499.1:75635351 | LOC120777560 | NC_052499.1 (75570372..75641059) | cadherin-99C | promotes cell adhesion and enhances cellular stability and integrity as a protective mechanism against heat stress in the thermophilic ant genus *Cataglyphis*; confers resistance to Bt toxin in transgenic contton | (Gao et al., 2018; Perez et al., 2021) |
| Native vs. Pacific Islands | NC_052500.1:18467508 | LOC120768270 LOC120767202 | NC_052500.1 (18456542..18457851) NC_052500.1 (18459100..18475526) | protein YIPF6  trehalose-phosphate phosphatase B | transmemberane protein located in Golgi apparatus and ER likely involved in thermotolerance in soldier flies (Stratiomyidae) larvae | flybase (Garbuz et al., 2008) |
| Native vs. Pacific Islands | NC_052500.1:46309826 | no exons | NA | NA | NA |  |
| Native vs. Pacific Islands | NC_052500.1:60188524 | no exons | NA | NA | NA |  |
| Native vs. Pacific Islands | NC_052501.1:14700025 | LOC120771906 | NC_052501.1 (14690247..14693654) | endoplasmic reticulum junction formation protein lunapark-A | involved in network formation and organization of ER | flybase |
| Native vs. Pacific Islands | NC_052501.1:29609593 | LOC120771668 LOC120771468 LOC120772200 LOC120771738 LOC120771736 | NC_052501.1 (29597629..29601158) NC_052501.1 (29601740..29607598) NC_052501.1 (29608398..29610980) NC_052501.1 (29611085..29612889) NC_052501.1 (29613795..29672282) | ARF GTPase-activating protein GIT2  mannosylglucosyl-3-phosphoglycerate phosphatase conserved oligomeric Golgi complex subunit 7  uncharacterized  transcription factor GAGA | regulates intracellular traffic and involved in vesicle formation hydrolase activity involved in glycosylation (the process of sugar molecules to proteins) and retrograde vesicular trafficking involved in cell division and gametogenesis | (Spang et al., 2010); UniProt; (Quental et al., 2010); flybase |
| Native vs. Pacific Islands | NC_052501.1:86078491 | LOC120770152 LOC120770153 | NC_052501.1 (86040271..86081443) NC_052501.1 (86081444..86083781) | metabotropic glutamate receptor 2 maltase A1-like | involved in cold sensing behaviour (in *C. elegans*) mediates hydrolysis and carbohydrate metabolism, potentially involved in long-distance flight (was upregulated in migratory forms of hoverflies) | (Doyle et al., 2022; Gong et al., 2019) |
| Native vs. Pacific Islands | NC_052501.1:86202350 | LOC120772156 | NC_052501.1 (86173787..86197464) | COP9 signalosome complex subunit 7 | involved in social behaviour, immunity, and adult physiology | (Tong et al., 2015; Zhang et al., 2019) |
| Native vs. Pacific Islands | NC_052502.1:24557499 | LOC120775939 | NC_052502.1 (24375180..24831193) | low-density lipoprotein receptor-related protein 2 | up-regulated in response to fluralaner insecticide | (Jia et al., 2020) |
| Native vs. Pacific Islands | NC_052502.1:49154112 | LOC120774945 | NC_052502.1 (49157048..49158468) | odorant receptor 7a-like | enables detection of volatile chemicals | flybase |
| Native vs. Pacific Islands | NC_052502.1:53034145 | LOC120774360 | NC_052502.1 (53042562..53047202) | uncharacterized | NA |  |
| Native vs. Pacific Islands | NW_024395342.1:8297 | no exons | NA | NA | NA |  |
| Native vs. Pacific Islands | NW_024396573.1:540274 | no exons | NA | NA | NA |  |


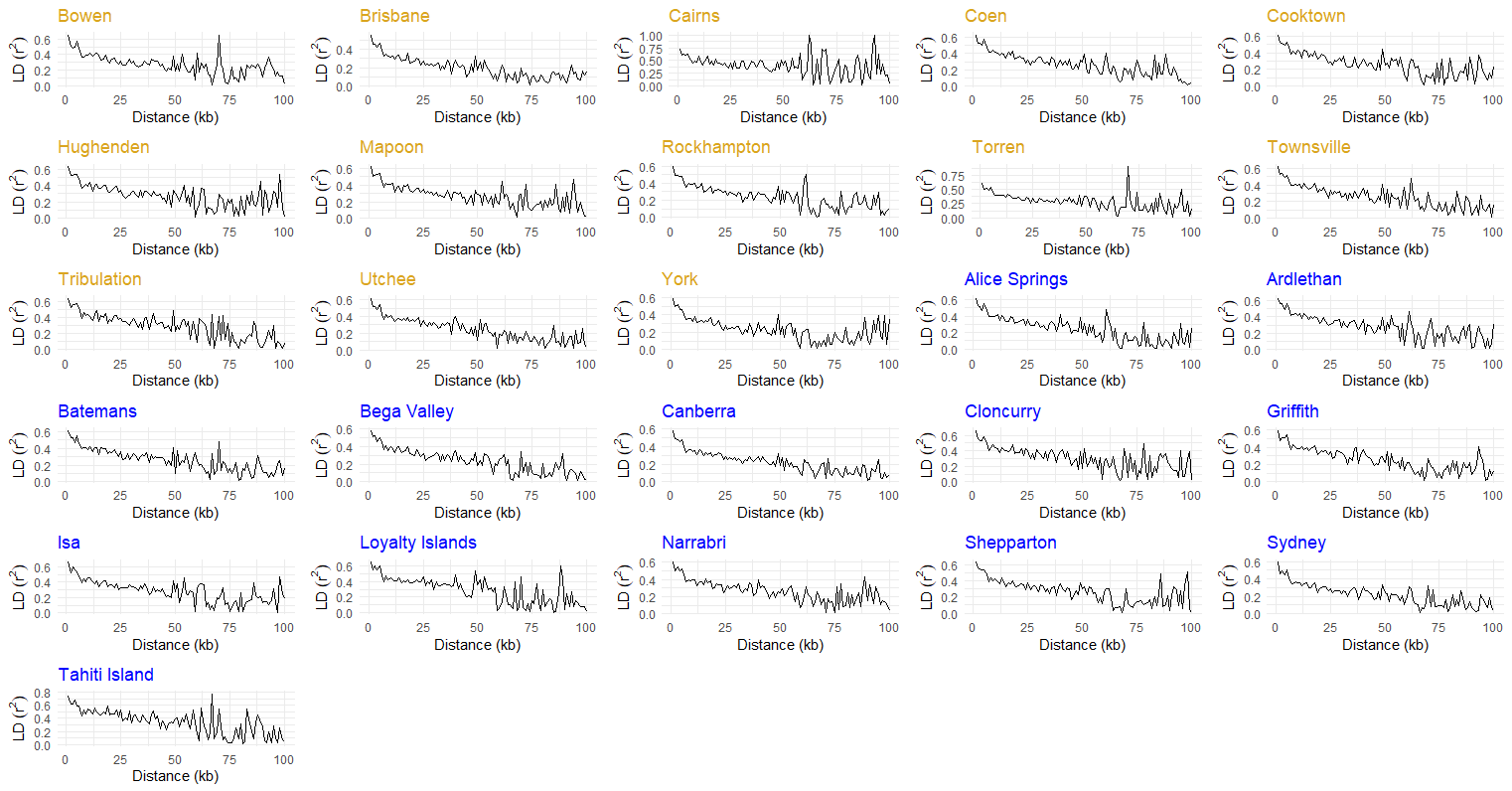


Figure S1. Patterns of linkage disequilibrium decay across the studied SNPs for native (yellow) and invasive (blue) populations of *Bactrocera tryoni*. This analysis was performed only on samples with n > 5.


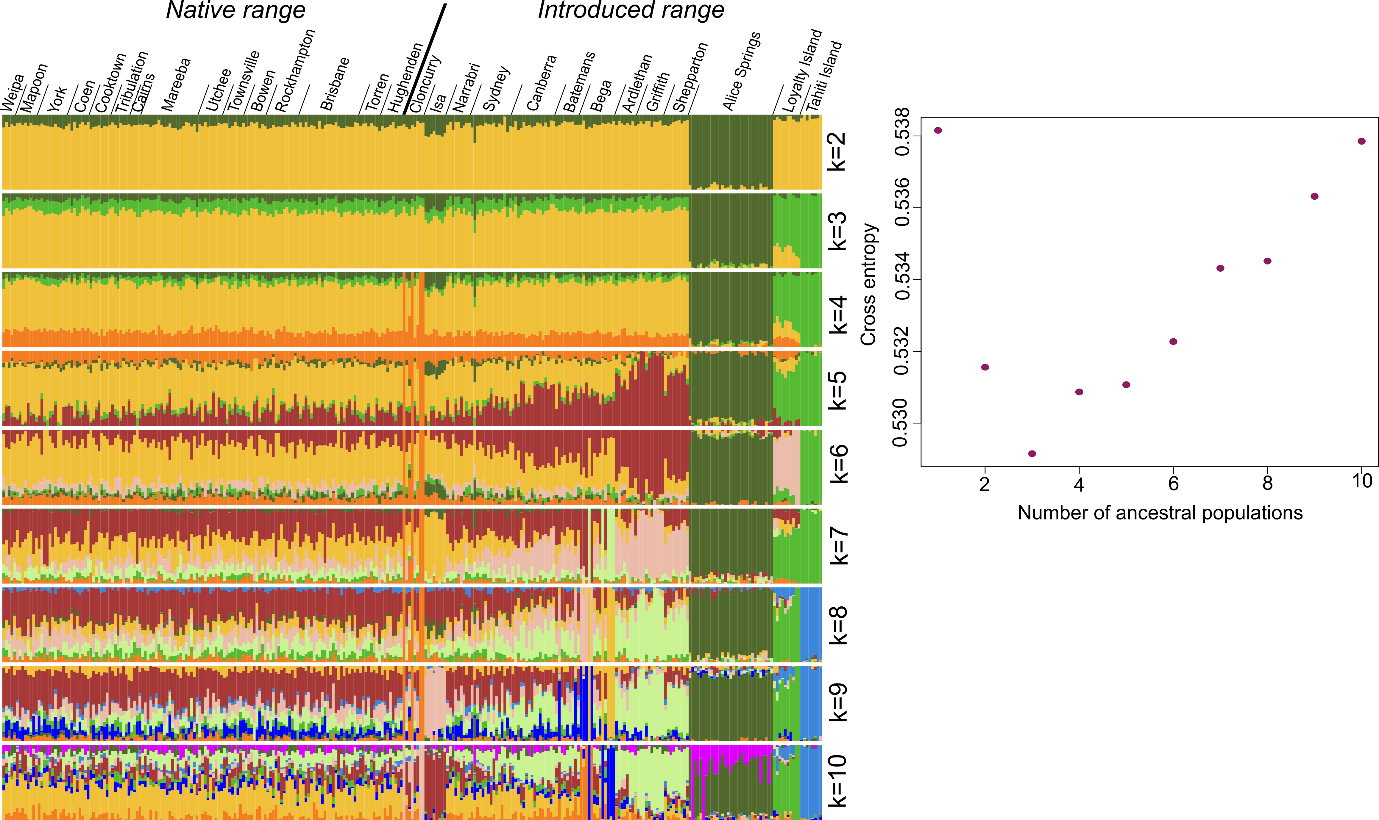


Figure S2. (a) Admixture plots obtained from sNMF analysis of native and introduced populations of *Bactrocera tryoni* based on 6,707 SNPs. Ancestry coefficients were estimated for k=1-10 and results are shown for k=2-10; (b) Cross-entropy results from sNMF analyses showing the optimal number of ancestral populations (i.e. k=3).


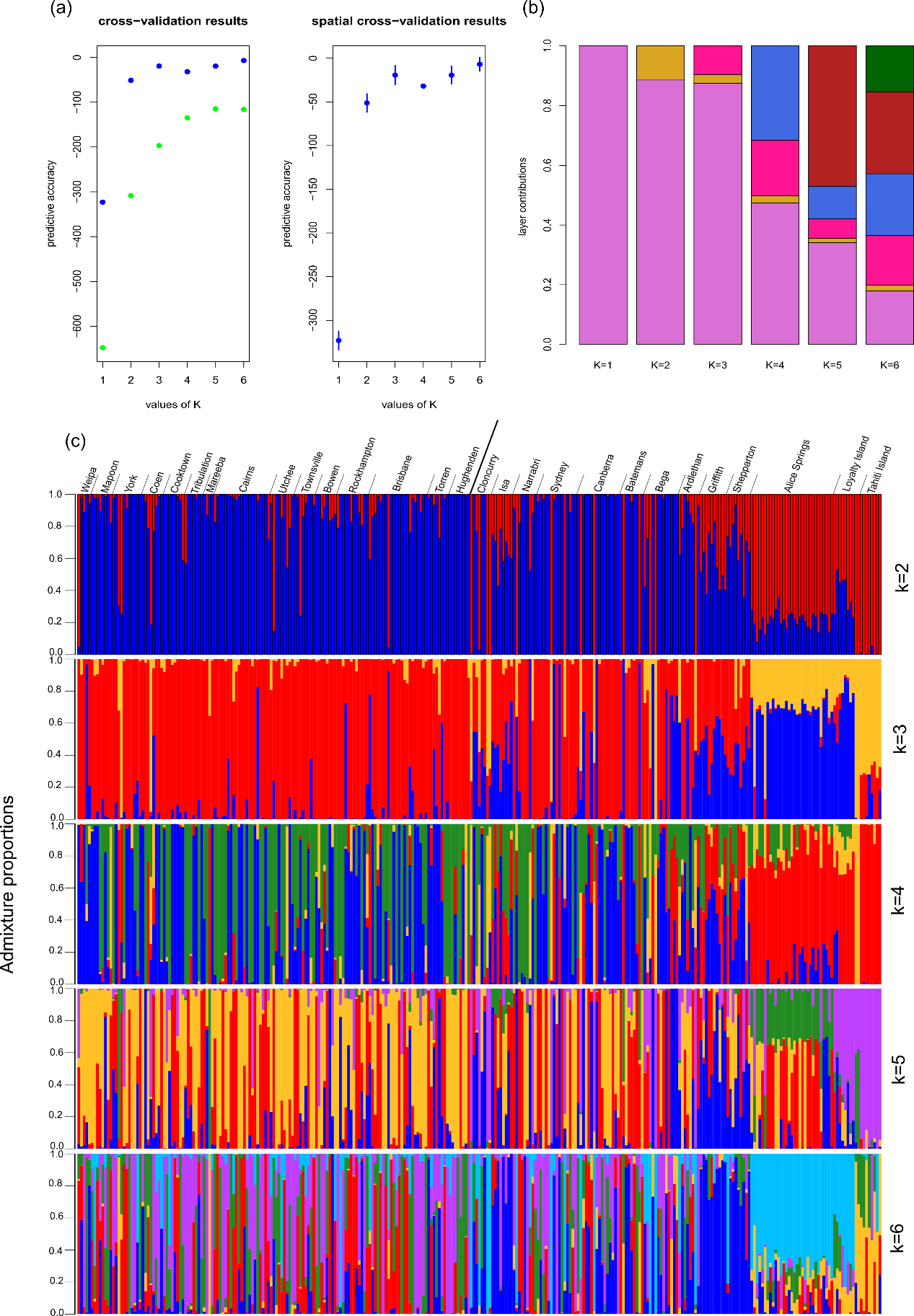


Figure S3. Inferring continuous and discrete population structure using conStruct analysis in *Bactrocera tryoni* based on 6,707 SNPs. (a) Cross-validation results under k=1-6, comparing the spatial (blue) and non-spatial (green) conStruct models. The spatial model showed better model fit at every value of k (i.e., predictive accuracy closer to zero); (b) Layer contribution results for k=1-6 of the spatial model. At k > 3, the smallest layer contribution was smaller than 2%; (c) Admixture proportions estimated using the spatial model for k=1-6.

# References

Ahlskog, J. K., Björk, J. K., Elsing, A. N., Aspelin, C., Kallio, M., Roos-Mattjus, P., & Sistonen, L. (2010). Anaphase-Promoting Complex/Cyclosome Participates in the Acute Response to Protein-Damaging Stress. *Molecular and Cellular Biology*, *30*(24), 5608–5620. https://doi.org/10.1128/MCB.01506-09

Blazek, E., Mittler, G., & Meisterernst, M. (2005). The Mediator of RNA polymerase II. *Chromosoma*, *113*(8), 399–408. https://doi.org/10.1007/s00412-005-0329-5

Bustos, F., Segarra-Fas, A., Nardocci, G., Cassidy, A., Antico, O., Davidson, L., Brandenburg, L., Macartney, T. J., Toth, R., Hastie, C. J., Moran, J., Gourlay, R., Varghese, J., Soares, R. F., Montecino, M., & Findlay, G. M. (2020). Functional Diversification of SRSF Protein Kinase to Control Ubiquitin-Dependent Neurodevelopmental Signaling. *Developmental Cell*, *55*(5), 629-647.e7. https://doi.org/10.1016/j.devcel.2020.09.025

Chown, S. L., Sørensen, J. G., & Terblanche, J. S. (2011). Water loss in insects: An environmental change perspective. *Journal of Insect Physiology*, *57*(8), 1070–1084. https://doi.org/10.1016/j.jinsphys.2011.05.004

Crossley, M. S., Chen, Y. H., Groves, R. L., & Schoville, S. D. (2017). Landscape genomics of Colorado potato beetle provides evidence of polygenic adaptation to insecticides. *Molecular Ecology*, *26*(22), 6284–6300. https://doi.org/10.1111/mec.14339

De Vos, M., Denekamp, M., Dicke, M., Vuylsteke, M., Van Loon, L., Smeekens, S. C., & Pieterse, C. (2006). The Arabidopsis thaliana Transcription Factor AtMYB102 Functions in Defense Against The Insect Herbivore Pieris rapae. *Plant Signaling & Behavior*, *1*(6), 305–311. https://doi.org/10.4161/psb.1.6.3512

Dennenmoser, S., Vamosi, S. M., Nolte, A. W., & Rogers, S. M. (2017). Adaptive genomic divergence under high gene flow between freshwater and brackish-water ecotypes of prickly sculpin (Cottus asper) revealed by Pool-Seq. *Molecular Ecology*, *26*(1), 25–42. https://doi.org/10.1111/mec.13805

Dong, K. (2007). Insect sodium channels and insecticide resistance. *Invertebrate Neuroscience : IN*, *7*(1), 17–30. https://doi.org/10.1007/s10158-006-0036-9

Doyle, T., Jimenez-Guri, E., Hawkes, W. L. S., Massy, R., Mantica, F., Permanyer, J., Cozzuto, L., Hermoso Pulido, T., Baril, T., Hayward, A., Irimia, M., Chapman, J. W., Bass, C., & Wotton, K. R. (2022). Genome-wide transcriptomic changes reveal the genetic pathways involved in insect migration. *Molecular Ecology*, *31*(16), 4332–4350. https://doi.org/10.1111/mec.16588

Gáliková, M., Dircksen, H., & Nässel, D. R. (2018). The thirsty fly: Ion transport peptide (ITP) is a novel endocrine regulator of water homeostasis in Drosophila. *PLOS Genetics*, *14*(8), e1007618. https://doi.org/10.1371/journal.pgen.1007618

Gao, M., Wang, X., Yang, Y., Tabashnik, B. E., & Wu, Y. (2018). Epistasis confers resistance to Bt toxin Cry1Ac in the cotton bollworm. *Evolutionary Applications*, *11*(5), 809–819. https://doi.org/10.1111/eva.12598

Garbuz, D. G., Zatsepina, O. G., Przhiboro, A. A., Yushenova, I., Guzhova, I. V., & Evgen’ev, M. B. (2008). Larvae of related Diptera species from thermally contrasting habitats exhibit continuous up-regulation of heat shock proteins and high thermotolerance. *Molecular Ecology*, *17*(21), 4763–4777. https://doi.org/10.1111/j.1365-294X.2008.03947.x

Garcia, M., Ray, S., Brown, I., Irom, J., & Brazill, D. (2014). PakD, a Putative p21-Activated Protein Kinase in Dictyostelium discoideum, Regulates Actin. *Eukaryotic Cell*, *13*(1), 119–126. https://doi.org/10.1128/EC.00216-13

Garcia-Elfring, A., Paccard, A., Thurman, T. J., Wasserman, B. A., Palkovacs, E. P., Hendry, A. P., & Barrett, R. D. H. (2021). Using seasonal genomic changes to understand historical adaptation to new environments: Parallel selection on stickleback in highly-variable estuaries. *Molecular Ecology*, *30*(9), 2054–2064. https://doi.org/10.1111/mec.15879

Gong, J., Liu, J., Ronan, E. A., He, F., Cai, W., Fatima, M., Zhang, W., Lee, H., Li, Z., Kim, G.-H., Pipe, K. P., Duan, B., Liu, J., & Xu, X. Z. S. (2019). A Cold-Sensing Receptor Encoded by a Glutamate Receptor Gene. *Cell*, *178*(6), 1375-1386.e11. https://doi.org/10.1016/j.cell.2019.07.034

Gu, X., Zhao, Y., Su, Y., Wu, J., Wang, Z., Hu, J., Liu, L., Zhao, Z., Hoffmann, A. A., Chen, B., & Li, Z. (2019). A transcriptional and functional analysis of heat hardening in two invasive fruit fly species, Bactrocera dorsalis and Bactrocera correcta. *Evolutionary Applications*, *12*(6), 1147–1163. https://doi.org/10.1111/eva.12793

Guo, H., Wang, L., Wang, C., Guo, D., Xu, B., Guo, X., & Li, H. (2021). Identification of an Apis cerana zinc finger protein 41 gene and its involvement in the oxidative stress response. *Archives of Insect Biochemistry and Physiology*, *108*(1), e21830. https://doi.org/10.1002/arch.21830

Jia, Z.-Q., Liu, D., Peng, Y.-C., Han, Z.-J., Zhao, C.-Q., & Tang, T. (2020). Identification of transcriptome and fluralaner responsive genes in the common cutworm Spodoptera litura Fabricius, based on RNA-seq. *BMC Genomics*, *21*(1), 120. https://doi.org/10.1186/s12864-020-6533-0

Jones, C. M., Papanicolaou, A., Mironidis, G. K., Vontas, J., Yang, Y., Lim, K. S., Oakeshott, J. G., Bass, C., & Chapman, J. W. (2015). Genomewide transcriptional signatures of migratory flight activity in a globally invasive insect pest. *Molecular Ecology*, *24*(19), 4901–4911. https://doi.org/10.1111/mec.13362

Kim, S., Kim, K., Lee, J. H., Han, S. H., & Lee, S. H. (2019). Differential expression of acetylcholinesterase 1 in response to various stress factors in honey bee workers. *Scientific Reports*, *9*(1), 1. https://doi.org/10.1038/s41598-019-46842-0

King, A. M., & MacRae, T. H. (2015). Insect heat shock proteins during stress and diapause. *Annual Review of Entomology*, *60*, 59–75. https://doi.org/10.1146/annurev-ento-011613-162107

Kirk, N. L., Howells, E. J., Abrego, D., Burt, J. A., & Meyer, E. (2018). Genomic and transcriptomic signals of thermal tolerance in heat-tolerant corals (Platygyra daedalea) of the Arabian/Persian Gulf. *Molecular Ecology*, *27*(24), 5180–5194. https://doi.org/10.1111/mec.14934

Kusakabe, M., Ishikawa, A., Ravinet, M., Yoshida, K., Makino, T., Toyoda, A., Fujiyama, A., & Kitano, J. (2017). Genetic basis for variation in salinity tolerance between stickleback ecotypes. *Molecular Ecology*, *26*(1), 304–319. https://doi.org/10.1111/mec.13875

Li, J.-J., Shi, Y., Lin, G.-L., Yang, C.-H., & Liu, T.-X. (2021). Genome-wide identification of neuropeptides and their receptor genes in Bemisia tabaci and their transcript accumulation change in response to temperature stresses. *Insect Science*, *28*(1), 35–46. https://doi.org/10.1111/1744-7917.12751

Ma, W.-J., Pannebakker, B. A., Li, X., Geuverink, E., Anvar, S. Y., Veltsos, P., Schwander, T., van de Zande, L., & Beukeboom, L. W. (2021). A single QTL with large effect is associated with female functional virginity in an asexual parasitoid wasp. *Molecular Ecology*, *30*(9), 1979–1992. https://doi.org/10.1111/mec.15863

Maxwell, B. A., Gwon, Y., Mishra, A., Peng, J., Nakamura, H., Zhang, K., Kim, H. J., & Taylor, J. P. (2021). Ubiquitination is essential for recovery of cellular activities after heat shock. *Science*, *372*(6549), eabc3593. https://doi.org/10.1126/science.abc3593

Mayfield, A. B., Wang, Y.-B., Chen, C.-S., Lin, C.-Y., & Chen, S.-H. (2014). Compartment-specific transcriptomics in a reef-building coral exposed to elevated temperatures. *Molecular Ecology*, *23*(23), 5816–5830. https://doi.org/10.1111/mec.12982

Mishra, A. K., Fritsch, C., Voutev, R., Mann, R. S., & Sprecher, S. G. (2021). Homothorax controls a binary Rhodopsin switch in Drosophila ocelli. *PLOS Genetics*, *17*(7), e1009460. https://doi.org/10.1371/journal.pgen.1009460

Morton, D. B., & Vermehren, A. (2007). Soluble guanylyl cyclases in invertebrates: Targets for NO and O2. In B. Tota & B. Trimmer (Eds.), *Advances in Experimental Biology* (Vol. 1, pp. 65–82). Elsevier. https://doi.org/10.1016/S1872-2423(07)01003-4

Ogura, T., Tan, A., Tsubota, T., Nakakura, T., & Shiotsuki, T. (2009). Identification and Expression Analysis of Ras Gene in Silkworm, Bombyx mori. *PLOS ONE*, *4*(11), e8030. https://doi.org/10.1371/journal.pone.0008030

Pauls, D., Hamarat, Y., Trufasu, L., Schendzielorz, T. M., Gramlich, G., Kahnt, J., Vanselow, J. T., Schlosser, A., & Wegener, C. (2019). Drosophila carboxypeptidase D (SILVER) is a key enzyme in neuropeptide processing required to maintain locomotor activity levels and survival rate. *European Journal of Neuroscience*, *50*(9), 3502–3519. https://doi.org/10.1111/ejn.14516

Perez, R., de Souza Araujo, N., Defrance, M., & Aron, S. (2021). Molecular adaptations to heat stress in the thermophilic ant genus Cataglyphis. *Molecular Ecology*, *30*(21), 5503–5516. https://doi.org/10.1111/mec.16134

Pitsouli, C., & Perrimon, N. (2013). The Homeobox Transcription Factor Cut Coordinates Patterning and Growth During Drosophila Airway Remodeling. *Science Signaling*, *6*(263), ra12–ra12. https://doi.org/10.1126/scisignal.2003424

Popa-Báez, Á.-D., Catullo, R., Lee, S. F., Yeap, H. L., Mourant, R. G., Frommer, M., Sved, J. A., Cameron, E. C., Edwards, O. R., Taylor, P. W., & Oakeshott, J. G. (2020). Genome-wide patterns of differentiation over space and time in the Queensland fruit fly. *Scientific Reports*, *10*(1), 10788. https://doi.org/10.1038/s41598-020-67397-5

Qin, G., Hu, X., Cebe, P., & Kaplan, D. L. (2012). Mechanism of resilin elasticity. *Nature Communications*, *3*(1), 1. https://doi.org/10.1038/ncomms2004

Quental, R., Azevedo, L., Matthiesen, R., & Amorim, A. (2010). Comparative analyses of the Conserved Oligomeric Golgi (COG) complex in vertebrates. *BMC Evolutionary Biology*, *10*(1), 212. https://doi.org/10.1186/1471-2148-10-212

Raghupathi Reddy, S. R., & Campbell, J. W. (1969). Arginine metabolism in insects. Role of arginase in proline formation during silkmoth development. *The Biochemical Journal*, *115*(3), 495–503. https://doi.org/10.1042/bj1150495

Ren, G. R., Hauser, F., Rewitz, K. F., Kondo, S., Engelbrecht, A. F., Didriksen, A. K., Schjøtt, S. R., Sembach, F. E., Li, S., Søgaard, K. C., Søndergaard, L., & Grimmelikhuijzen, C. J. P. (2015). CCHamide-2 Is an Orexigenic Brain-Gut Peptide in Drosophila. *PLOS ONE*, *10*(7), e0133017. https://doi.org/10.1371/journal.pone.0133017

Rösner, J., Tietmeyer, J., & Merzendorfer, H. (2021). Organic anion-transporting polypeptides are involved in the elimination of insecticides from the red flour beetle, Tribolium castaneum. *Journal of Pest Science*, *94*(4), 1427–1437. https://doi.org/10.1007/s10340-020-01317-4

Spang, A., Shiba, Y., & Randazzo, P. A. (2010). ArfGAPs: Gatekeepers of vesicle generation. *FEBS Letters*, *584*(12), 2646–2651. https://doi.org/10.1016/j.febslet.2010.04.005

Stengl, M., & Funk, N. W. (2013). The role of the coreceptor Orco in insect olfactory transduction. *Journal of Comparative Physiology A*, *199*(11), 897–909. https://doi.org/10.1007/s00359-013-0837-3

Tauber, E., Zordan, M., Sandrelli, F., Pegoraro, M., Osterwalder, N., Breda, C., Daga, A., Selmin, A., Monger, K., Benna, C., Rosato, E., Kyriacou, C. P., & Costa, R. (2007). Natural Selection Favors a Newly Derived timeless Allele in Drosophila melanogaster. *Science*, *316*(5833), 1895–1898. https://doi.org/10.1126/science.1138412

Tong, X.-W., Chen, B., Huang, L.-H., Feng, Q.-L., & Kang, L. (2015). Proteomic analysis reveals that COP9 signalosome complex subunit 7A (CSN7A) is essential for the phase transition of migratory locust. *Scientific Reports*, *5*(1), 1. https://doi.org/10.1038/srep12542

Tucker, N. R., & Shelden, E. A. (2009). Hsp27 associates with the titin filament system in heat-shocked zebrafish cardiomyocytes. *Experimental Cell Research*, *315*(18), 3176–3186. https://doi.org/10.1016/j.yexcr.2009.06.030

Uyhelji, H. A., Cheng, C., & Besansky, N. J. (2016). Transcriptomic differences between euryhaline and stenohaline malaria vector sibling species in response to salinity stress. *Molecular Ecology*, *25*(10), 2210–2225. https://doi.org/10.1111/mec.13609

Vargas, S., Zimmer, T., Conci, N., Lehmann, M., & Wörheide, G. (2022). Transcriptional response of the calcification and stress response toolkits in an octocoral under heat and pH stress. *Molecular Ecology*, *31*(3), 798–810. https://doi.org/10.1111/mec.16266

Vatanparast, M., Puckett, R. T., Choi, D.-S., & Park, Y. (2021). Comparison of gene expression in the red imported fire ant (Solenopsis invicta) under different temperature conditions. *Scientific Reports*, *11*(1), 1. https://doi.org/10.1038/s41598-021-95779-w

von Wyschetzki, K., Rueppell, O., Oettler, J., & Heinze, J. (2015). Transcriptomic Signatures Mirror the Lack of the Fecundity/Longevity Trade-Off in Ant Queens. *Molecular Biology and Evolution*, *32*(12), 3173–3185. https://doi.org/10.1093/molbev/msv186

Wiche, G. (1998). Role of plectin in cytoskeleton organization and dynamics. *Journal of Cell Science*, *111*(17), 2477–2486. https://doi.org/10.1242/jcs.111.17.2477

Zhang, J., Zhang, Z., Zhang, R., Zhang, W., Li, H., Li, T., Zhang, H., & Zheng, W. (2019). Identification of COP9 Signalosome Subunit Genes in Bactrocera dorsalis and Functional Analysis of csn3 in Female Fecundity. *Frontiers in Physiology*, *10*, 162. https://doi.org/10.3389/fphys.2019.00162
